# The Gut-Brain Homeostatic Continuum in Alzheimer’s disease: a systems framework for physiological resilience and disease progression

**DOI:** 10.3389/fnins.2026.1963040

**Published:** 2026-10-19

**Authors:** Muriel Jacquier-Sarlin, Maxime Seignobos, Louis Rauzier, Béatrice Schaack, David Laurin, Alain Buisson

**Affiliations:** 1 Univ. Grenoble Alpes, Institut National de la Santé et de la Recherche Médicale (INSERM) U1216, Grenoble Institut Neurosciences (GIN), Grenoble, France; 2 National Centre for Scientific Research (CNRS), Unité Mixte de Recherche (UMR) 5525, Translational Research and Innovation in Medicine and Complexity (TIMC), University Grenoble Alpes, Grenoble, France; 3 Commissariat à l’Énergie Atomique (CEA), National Centre for Scientific Research (CNRS), Unité Mixte de Recherche (UMR) 5075, Institut de Biologie Structurale (IBS), University Grenoble Alpes, Grenoble, France; 4 Institute for Advanced Biosciences, Institut National de la Santé et de la Recherche Médicale (INSERM) U1209, National Centre for Scientific Research (CNRS) and Etablissement Français Du Sang (EFS), Unité Mixte de Recherche (UMR) 5309, Grenoble, France

**Keywords:** Alzheimer disease, brain-gut axis, clearance, extracellular vesicles, gastrointestinal microbiome, homeostasis, neuroinflammation, proteostasis

## Abstract

The gut-brain axis has substantially improved our understanding of Alzheimer’s disease (AD) by highlighting the complex bidirectional interactions between the gastrointestinal tract, the microbiota and the central nervous system. Nevertheless, this framework does not fully explain why brain and organismal homeostasis can be maintained for decades despite the continuous and cumulative burden of aging, genetic susceptibility and environmental stressors, or why this resilience ultimately fails in some individuals. In this review, we propose the Gut-Brain Homeostatic Continuum (GBHC), a conceptual framework that extends the classical gut-brain axis into a dynamic, interconnected system of layered homeostatic mechanisms. Rather than viewing gut-brain interactions as parallel signaling routes, the GBHC emphasizes how the preservation of intestinal and blood-brain barrier integrity supports coordinated immune, metabolic and inter-organ regulation, which in turn contributes to systemic proteostasis and the trafficking and clearance of proteins. Through these interconnected processes, peripheral physiological states may influence the capacity of the brain to maintain homeostasis and resilience. Within this framework, AD is viewed as a progressive erosion of organismal resilience rather than the consequence of a single pathological pathway. We discuss how aging, genetic factors and the exposome may progressively challenge interconnected regulatory mechanisms, weakening the capacity to preserve homeostasis and increasing vulnerability to neurodegeneration. This perspective also suggests a complementary approach to biomarker discovery, focusing on multimodal signatures that capture biological trajectories and changes in regulatory capacity. Such signatures could integrate markers of barrier integrity and permeability, immune and metabolic regulation, microbial and host-derived metabolites, extracellular vesicle cargo, and proteostasis-related proteins, including established markers of neurodegeneration such as amyloid-β, phosphorylated tau and neurofilament light chain. Although several aspects of the GBHC remain hypothetical and require experimental validation, this framework provides an integrative model that complements current concepts of AD pathogenesis and opens new perspectives for multidisciplinary research and therapeutic innovation.

## 1 Introduction

### 1.1 Alzheimer’s disease: from brain pathology to a broader biological perspective

Alzheimer’s disease (AD) is the most common cause of dementia and represents a major and growing public health challenge worldwide. In 2019, an estimated 57 million people worldwide were living with dementia, a number projected to reach 153 million by 2050 (Nichols et al., 2022), emphasizing the urgent need for effective prevention strategies. AD is characterized neuropathologically by the accumulation of amyloid-β (Aβ) plaques and hyperphosphorylated tau aggregates, accompanied by synaptic dysfunction, neuroinflammation, neuronal loss and progressive brain atrophy (Jellinger, 2020; Leng and Edison, 2021; Tzioras et al., 2023). Advances in biomarkers, including cerebrospinal fluid (CSF) and plasma assays, molecular imaging, and more recently, disease-modifying therapies targeting amyloid pathology (Scheltens et al., 2021; Rafii and Aisen, 2025; Kobayashi et al., 2026), have further established that these pathological changes can develop many years before the emergence of overt cognitive symptoms (Sperling et al., 2011). The biological definition of AD has therefore increasingly shifted from a purely clinical diagnosis toward the identification of distinct pathological processes using biomarkers of amyloid, tau and neurodegeneration (Jack et al., 2018).

Yet, AD remains an unresolved biological paradox. A substantial proportion of cognitively unimpaired older individuals can exhibit significant amyloid pathology, and in some cases, early tau pathology, while showing limited or no clinical impairment (Aizenstein et al., 2008; Ziontz et al., 2019). Conversely, individuals with apparently comparable levels of neuropathological burden may exhibit markedly different degrees of cognitive dysfunction, a discrepancy often discussed in relation to cognitive reserve and resilience (Stern, 2012; Stern et al., 2019). Although aging remains the strongest risk factor for sporadic AD and dementia, dementia is not an inevitable consequence of advanced age (Qiu and Fratiglioni, 2018). The accumulation of disease-associated pathology therefore does not translate into a uniform functional outcome. These observations suggest that cerebral pathology alone may not be sufficient to fully explain individual differences in disease susceptibility, progression and clinical expression.

This discrepancy raises a fundamental question: What enables some individuals to maintain physiological and cognitive function despite prolonged exposure to biological stressors, whereas others progressively lose this capacity? Increasing evidence indicates that aging, genetic susceptibility, vascular and metabolic alterations, immune dysregulation, environmental exposures and lifestyle factors can influence both the accumulation of AD-related pathology and its clinical expression. The brain therefore develops and responds to pathological processes within a broader physiological context in which multiple interconnected systems contribute to tissue integrity, metabolic balance, immune regulation and protein homeostasis. Understanding AD may consequently require a shift from a predominantly brain-centered perspective toward a more integrated view of the biological systems that influence vulnerability and resilience over time.

### 1.2 The evolution of complementary frameworks in Alzheimer’s disease

Over the past several decades, multiple conceptual frameworks have progressively expanded the biological landscape of AD. The amyloid cascade framework emerged from the identification and characterization of Aβ in the 1980s and its subsequent formulation as a central pathogenic event in AD (Glenner and Wong, 1984; Masters et al., 1985; Hardy and Higgins, 1992; Karran and De Strooper, 2016). Tau-centered models subsequently emphasized the relationship between tau pathology, neurodegeneration, anatomical progression and cognitive decline (Braak and Braak, 1991; Iqbal et al., 2005). Neuroimmune frameworks highlighted the contribution of microglial responses, innate immunity and inflammatory signaling, supported in part by genetic evidence implicating immune pathways in AD susceptibility and progression (Akiyama et al., 2000; Ulland and Colonna, 2018; Leng and Edison, 2021). In parallel, vascular and blood-brain barrier (BBB) models emphasized cerebrovascular dysfunction, neurovascular regulation and barrier integrity as determinants of cerebral vulnerability (de la Torre, 1994; Zlokovic, 2011; Sweeney et al., 2018). Metabolic and mitochondrial perspectives further broadened the field by implicating glucose and insulin signaling, lipid metabolism, mitochondrial dysfunction and oxidative stress in neuronal and systemic vulnerability (Swerdlow and Khan, 2004; de la Monte and Wands, 2006; De Felice et al., 2009; Bomfim et al., 2012; Wang et al., 2017; Arnold et al., 2018).

Systems-biology and network-based approaches have provided conceptual and analytical tools for examining how these mechanisms interact across multiple biological scales (Rosario et al., 2020; Altmann et al., 2024). Brain reserve, cognitive reserve and resilience are related but distinct concepts that have been developed to account for interindividual differences in the clinical expression of neuropathology (Stern, 2002, 2012; Montine et al., 2019; Stern et al., 2019; Bartrés-Faz et al., 2020; Bocancea et al., 2021).

More recently, the microbiota-gut-brain axis has introduced the gastrointestinal tract (GIT) and its microbial ecosystem into this broader biological landscape (Cattaneo et al., 2017; Dinan and Cryan, 2017). The gut-brain axis describes bidirectional communication between the GIT and the central nervous system (CNS) through neural, endocrine, immune and metabolic pathways (Cryan et al., 2019; Morais et al., 2021). The gut microbiota contributes to immune maturation, intestinal barrier integrity, bile acid metabolism and the production of bioactive metabolites, including short-chain fatty acids (SCFAs), tryptophan derivatives and secondary bile acids (Dalile et al., 2019). These microbial products may influence intestinal and systemic physiology, vascular function and BBB integrity, while the brain reciprocally regulates gastrointestinal motility, secretion and permeability through autonomic and neuroendocrine pathways. In AD, application of the gut-brain axis concept has revealed associations between alterations of the gut microbiota and several features of disease biology, including chronic inflammation, intestinal barrier dysfunction, altered microbial metabolism, neuroinflammation and cognitive impairment (Vogt et al., 2017; Manocha et al., 2019; Chen et al., 2020; Kim et al., 2020; Tarawneh and Penhos, 2022; Bello-Corral et al., 2023; Grabrucker et al., 2023; Lei et al., 2025). Resilience-oriented gut-immune-brain frameworks have further extended this perspective by emphasizing coordinated interactions among the microbiome, immune system, biological barriers and brain resilience during aging. The Gut-Immune-Brain Resilience Axis (GIBRA), for example, conceptualizes microbial signaling, immune regulation and barrier integrity as interconnected determinants of neuroimmune resilience and proposes functional microbiome endotyping as a basis for biomarker discovery and precision approaches (Bajić et al., 2026). These different perspectives should not be considered mutually exclusive or as successive models in which one explanation replaces another. Rather, they describe complementary components of a complex biological system, ranging from molecular pathology within the brain to vascular, metabolic, immune and peripheral processes. The evolution of these frameworks therefore reflects a progressive expansion of the biological landscape of AD, from disease-specific brain pathology toward increasingly interconnected views of organismal physiology and vulnerability (Figure 1).

**FIGURE 1:**
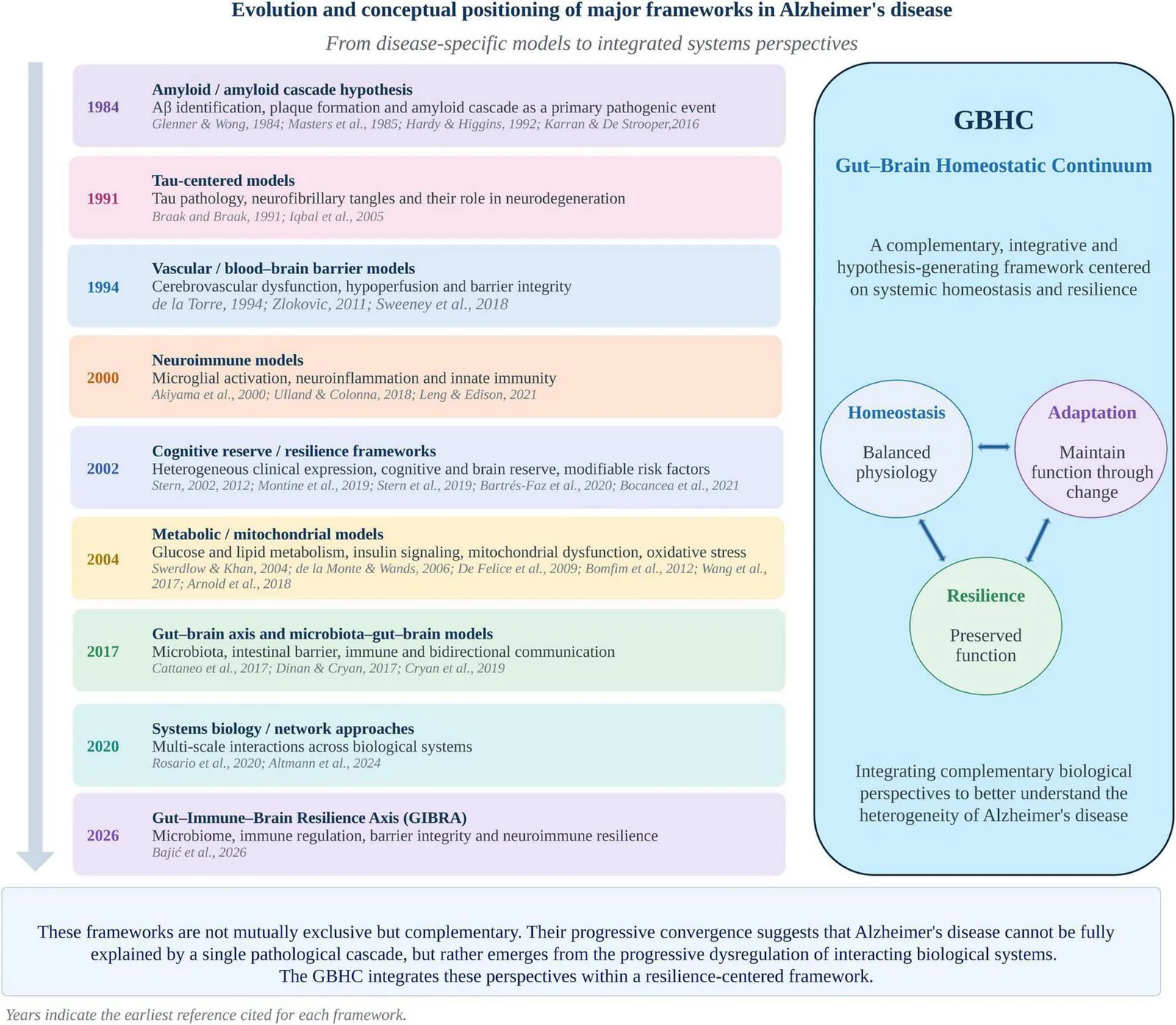
Evolution and conceptual positioning of major frameworks in Alzheimer’s disease. Conceptual evolution of major frameworks used to understand Alzheimer’s disease (AD), illustrating the progressive expansion from disease-specific molecular and brain-centered models toward increasingly interconnected perspectives of brain–body physiology. Amyloid- and tau-centered models focus on core molecular features of AD pathology, while neuroimmune, vascular and metabolic frameworks highlight additional biological processes contributing to disease vulnerability and progression. The gut-brain axis and microbiota–gut–brain models further incorporate bidirectional communication between the gastrointestinal tract, the microbiome and the brain. Systems-biology approaches emphasize interactions across biological networks and multiple biological scales, whereas brain and cognitive reserve and resilience frameworks address inter-individual differences in the capacity to preserve function despite accumulating pathology. Recent resilience-oriented approaches, including the Gut-Immune-Brain Resilience Axis (GIBRA), integrate gut, immune and barrier function with neuroimmune resilience. Within this evolving landscape, the Gut-Brain Homeostatic Continuum (GBHC) is proposed as a complementary, integrative and hypothesis-generating framework that brings together these interconnected biological domains around the concepts of homeostasis, adaptation, compensation and physiological resilience. The figure illustrates conceptual convergence rather than a succession of mutually exclusive models or a linear causal sequence of AD pathogenesis.

### 1.3 Conceptual positioning of the GBHC

The Gut-Brain Homeostatic Continuum (GBHC) builds on, rather than replaces, these existing frameworks. Its conceptual contribution is not to propose an additional biological pathway, but to provide an organizing perspective through which their complementary contributions can be considered within a dynamic trajectory of homeostasis, adaptation and resilience.

Conceptually, the GBHC places particular emphasis on three complementary dimensions. First, whereas the gut-brain axis primarily describes bidirectional communication between the gastrointestinal tract (GIT) and the brain, the GBHC places the maintenance and adaptation of homeostasis at the center of the framework. Second, whereas microbiota-gut-brain models emphasize microbial communities, metabolites and microbial-derived signals, the GBHC considers the microbiota as one component of a broader network of interacting physiological regulators. Third, whereas systems-biology approaches primarily characterize interactions across biological networks and scales, the GBHC emphasizes their potential evolution over time in relation to homeostatic maintenance, adaptation, compensation and progressive loss of resilience (Table 1).

**TABLE 1:** Evolution and conceptual positioning of major frameworks in Alzheimer’s disease.

Conceptual framework	Main focus	Main contribution to AD research	Remaining limitation/rationale for further integration	Key references
Amyloid cascade hypothesis	Amyloid-β (Aβ) accumulation and plaque formation	Positioned Aβ accumulation as a major pathological event and provided a mechanistic rationale for amyloid-targeting strategies	Does not fully explain clinical heterogeneity, the dissociation between amyloid burden and cognitive status, or the contribution of non-amyloid biological systems	Glenner and Wong, 1984; Masters et al., 1985; Hardy and Higgins, 1992; Karran and De Strooper, 2016
Tau-centered models	Tau pathology, neurofibrillary degeneration and spatial progression	Highlighted the strong relationship between tau pathology, neurodegeneration, anatomical progression and cognitive decline	Tau pathology alone does not capture the broader biological context in which pathology emerges and progresses	Braak and Braak, 1991; Iqbal et al., 2005
Vascular/blood–brain barrier models	Cerebrovascular dysfunction, neurovascular unit and BBB integrity	Established vascular dysfunction and altered barrier function as important determinants of brain vulnerability and AD pathology	Primarily focuses on the cerebral vascular compartment and does not by itself capture systemic inter-organ regulation	de la Torre, 1994; Zlokovic, 2011; Sweeney et al., 2018
Neuroimmune/microglial models	Microglial responses, innate immunity and neuroinflammation	Established neuroimmune mechanisms as important modulators of AD pathology and disease progression, including genetic evidence involving immune pathways	Immune responses are highly context- and stage-dependent and interact with peripheral, vascular and metabolic signals	Akiyama et al., 2000; Ulland and Colonna, 2018; Leng and Edison, 2021
Reserve/resilience frameworks	Inter-individual differences in response to neuropathology	Helped explain why comparable levels of neuropathology can be associated with markedly different clinical and cognitive outcomes	Traditionally centered on brain or cognitive reserve and less focused on coordinated adaptation across peripheral and central physiological systems	Stern, 2002, 2012; Montine et al., 2019; Stern et al., 2019; Bartrés-Faz et al., 2020; Bocancea et al., 2021
Metabolic/mitochondrial models	Glucose metabolism, insulin signaling, mitochondria, lipid metabolism and oxidative stress	Expanded AD biology beyond classical proteinopathies toward metabolic and bioenergetic dysfunction	Metabolic alterations are interconnected with immune, vascular, neuronal and peripheral mechanisms and are unlikely to constitute an isolated driver	Swerdlow and Khan, 2004; de la Monte and Wands, 2006; De Felice et al., 2009; Bomfim et al., 2012; Wang et al., 2017; Arnold et al., 2018
Gut–brain/microbiota–gut–brain axis	Bidirectional communication between the gut and brain	Introduced the gut microbiota, intestinal barrier, microbial metabolites, and immune, endocrine and neural pathways as potential modulators of brain physiology and AD-related processes	Primarily describes bidirectional communication rather than providing a general framework for how multiple physiological systems maintain homeostasis and resilience over time	Cattaneo et al., 2017; Dinan and Cryan, 2017; Cryan et al., 2019
Systems biology/network approaches	Multiscale interactions among biological systems	Emphasized the interconnected nature of genetic, molecular, metabolic, immune, vascular and neuronal processes	Captures biological complexity and interactions but does not necessarily provide a unifying physiological principle for how these systems adapt or lose resilience over time	Rosario et al., 2020; Altmann et al., 2024
Gut–Immune–Brain Resilience Axis (GIBRA)	Gut microbiome, immune regulation, barrier integrity and neuroimmune resilience during aging	Integrates microbial signaling, immune resilience and barrier function within an aging-related neuroimmune framework	More specifically centered on gut–immune–brain interactions and neuroimmune resilience; broader integration of systemic homeostasis, vascular/metabolic regulation, proteostasis and clearance remains to be developed	Bajić et al., 2026
**Gut–Brain Homeostatic Continuum (GBHC)**	**Dynamic systemic homeostasis, adaptation and resilience across interconnected organs and biological barriers**	**Integrates complementary biological perspectives within a temporal framework centered on the capacity of interconnected systems to maintain, adapt and restore physiological homeostasis**	**Conceptual framework proposed in this review; requires experimental and longitudinal validation and is not intended to replace established disease-specific models**	**This review**

AD, Alzheimer’s disease; Aβ, amyloid-β; BBB, blood–brain barrier; GIBRA, Gut–Immune–Brain Resilience Axis; GBHC, Gut–Brain Homeostatic Continuum. Bold text indicates the conceptual framework proposed in this review.

### 1.4 From gut-brain communication to systemic homeostasis

Within this evolving landscape, the gut-brain axis provides an important conceptual bridge between peripheral physiology and brain function. However, communication is not synonymous with homeostasis. The GBHC therefore extends this perspective by considering how interconnected organs, biological barriers, immune and metabolic systems, and protein trafficking and clearance mechanisms contribute to the maintenance and adaptation of physiological homeostasis over time. Physiological response to biological stress involves not only gut-brain signaling, but also interactions among the GIT, liver, kidneys and other peripheral organs, biological barriers, immune and metabolic systems, and mechanisms involved in protein trafficking, proteostasis and clearance. Biological barriers regulate selective molecular exchange and immune surveillance; peripheral organs participate in metabolism and physiological protein processing and clearance; microbial metabolites, immune mediators and extracellular vesicles (EVs) provide potential routes of biological communication between distant tissues; and protein trafficking contributes to systemic proteostasis. Putative inter-organ protein-trafficking pathways may also contribute to systemic proteostasis, although their existence and physiological relevance remain to be established. Together, these processes may form an interconnected homeostatic network capable of responding dynamically to physiological stress while contributing to the preservation of tissue integrity and organismal function.

This broader perspective is particularly relevant to AD because of its prolonged preclinical phase. The remarkable preservation of cognitive function in many individuals despite the progressive accumulation of amyloid pathology, tau aggregation and other biological alterations suggests that compensatory mechanisms may buffer perturbations for extended periods. Such compensation is unlikely to depend exclusively on a single organ or pathway. Rather, it may emerge from the coordinated activity of multiple interconnected systems that continuously respond to internal and environmental challenges.

### 1.5 From homeostasis to resilience

This broader perspective requires distinguishing between homeostasis, adaptation, resistance, reserve and resilience. Homeostasis refers to the maintenance of physiological conditions within functional ranges, whereas adaptive responses allow biological systems to respond dynamically to perturbations. Resistance generally refers to the capacity to limit or prevent functional or pathological changes in response to stress, whereas resilience refers to the capacity to maintain or recover function despite biological stress or accumulated pathology (Stern et al., 2020).

In the context of brain aging and AD, brain and cognitive resilience have been conceptualized as the capacity to preserve brain integrity or cognitive function despite accumulating age-related or AD-related pathology. These concepts complement the related frameworks of brain reserve and cognitive reserve (Bocancea et al., 2021; Ourry et al., 2026). Importantly, these frameworks have highlighted a fundamental dissociation between pathological burden and functional expression: individuals with similar levels of pathology may differ substantially in their ability to maintain functions. Within this context, the prolonged preclinical phase of AD may be interpreted not simply as a period of silent brain pathology, but also as a period during which compensatory mechanisms may remain sufficiently effective to preserve functions despite accumulating biological perturbations. These concepts are related but distinct: brain reserve generally refers to structural or quantitative properties of the brain, cognitive reserve to the ability to maintain cognitive performance despite brain pathology, and resilience to the capacity to preserve or recover function in the presence of biological stress or pathology. Within the GBHC, we use the term systemic resilience to refer to the proposed emergent property of interconnected peripheral and central physiological systems to maintain or recover homeostasis in response to cumulative biological perturbations. This concept extends the perspective of resilience beyond the brain without redefining established concepts of brain or cognitive resilience. We use the term systemic adaptive reserve to denote the hypothetical capacity of interconnected physiological systems to compensate for repeated perturbations while preserving organismal homeostasis.

### 1.6 The Gut–Brain Homeostatic Continuum: an integrative conceptual framework

The GBHC is proposed as a complementary, hypothesis-generating framework that organizes interconnected biological processes around a dynamic homeostatic perspective. Gut, barrier, immune, metabolic, vascular and neural processes are considered components of a broader physiological system whose capacity for adaptation and resilience may change over time. The framework extends existing gut-brain and neuroimmune perspectives by incorporating systemic homeostasis, inter-organ communication, proteostasis, and physiological protein trafficking and clearance, with particular relevance to the biological heterogeneity and trajectory of AD.

The framework emphasizes the trajectory of homeostatic maintenance, adaptation, compensation and progressive loss of resilience, rather than a single initiating mechanism or linear causal sequence. Four interconnected pillars are considered central: (i) the gut ecosystem, including the microbiota, intestinal epithelium and mucosal immune system; (ii) biological barriers, including the intestinal barrier (IB) and the BBB, which regulate selective molecular exchange and immune surveillance; (iii) bidirectional inter-organ communication through neural, immune, endocrine, metabolic and EV-mediated pathways; and (iv) proteostasis, protein trafficking and clearance involving the brain and peripheral organs.

The GBHC shifts the emphasis from identifying a single initiating mechanism toward understanding how biological processes interact within a dynamic physiological context. Aging, genetic susceptibility, environmental exposures, lifestyle and disease-related stressors may progressively challenge this network. In some individuals, compensatory mechanisms may preserve homeostasis, whereas reduced adaptive capacity may be associated with barrier dysfunction, altered inter-organ communication, chronic inflammation, impaired proteostasis, and disturbances in protein trafficking and clearance. Chronic immune activation may further contribute to AD pathogenesis (Karran and De Strooper, 2016; Long and Holtzman, 2019; Mary et al., 2024).

Importantly, the GBHC does not imply a linear or deterministic sequence from peripheral alterations to cerebral dysfunction or from systemic dysregulation to AD. The temporal relationships among gut, vascular, immune, metabolic and cerebral alterations are likely to be heterogeneous and bidirectional and may differ across individuals and disease stages. The GBHC should therefore be regarded as a hypothesis-generating model that organizes established biological mechanisms while providing testable hypotheses regarding their interactions and contribution to resilience (Figure 1). By providing a systems-level perspective, the GBHC does not seek to redefine AD pathology or establish a new causal sequence. Instead, it proposes an organizing framework in which established disease mechanisms can be considered according to their potential contribution to the maintenance or progressive disruption of systemic homeostasis. The framework thus connects disease-specific mechanisms with a broader question: how does an organism maintain functional resilience in the face of accumulating biological stress, and under what conditions might this adaptive capacity become insufficient?

### 1.7 Applicability to early-onset Alzheimer’s disease

The GBHC framework is primarily intended to describe the multifactorial trajectory of late-onset Alzheimer’s disease (LOAD), in which aging-related changes interact with genetic susceptibility and cumulative environmental, metabolic, vascular and immune stressors. Early-onset AD (EOAD), however, is biologically heterogeneous and should not be considered a single distinct entity. A subset of EOAD cases is caused by highly penetrant autosomal-dominant mutations in APP, PSEN1 or PSEN2, in which a strong genetic determinant can substantially shape the disease trajectory, whereas many EOAD cases are non-Mendelian and may share complex genetic and environmental determinants with late-onset AD (Mendez, 2019; Reitz et al., 2020). The GBHC may therefore be applicable across AD subtypes, although the relative contribution and temporal dynamics of homeostatic stressors and resilience mechanisms are likely to differ according to genetic and biological context.

Childhood-onset neurodegenerative disorders associated with Alzheimer-like phenotypes or pathology represent a further distinct category. Childhood-onset neurodegenerative disorders associated with dementia encompass a heterogeneous group of rare conditions, many of which are caused by genetic, metabolic, lysosomal, mitochondrial or other developmental mechanisms that differ substantially from those underlying adult-onset AD (Mastrangelo, 2019). Although some of these disorders may converge on mechanisms such as impaired proteostasis, mitochondrial dysfunction, oxidative stress, neuroinflammation or altered cellular homeostasis, they should not be considered equivalent to childhood-onset Alzheimer’s disease or simply extrapolated from the adult AD trajectory.

These disorders are acknowledged here but remain outside the primary scope of the present review, which focuses on the multifactorial biology of adult-onset AD.

### 1.8 Aim of the review

In this review, we develop the GBHC as a conceptual framework integrating evidence linking intestinal, vascular, immune, metabolic and cerebral physiology in AD. We examine the contribution of these systems to homeostasis and resilience, the mechanisms through which they communicate and become dysregulated, and the roles of biological barriers, extracellular vesicles, protein trafficking and clearance. Finally, we discuss testable hypotheses and research priorities for determining whether systemic homeostatic resilience can be operationalized, quantified and potentially modulated therapeutically in AD.

## 2 Building the Gut-Brain Homeostatic Continuum

The GBHC is organized around four complementary functional pillars that contribute to organismal homeostasis (Figure 2): (i) the gut ecosystem; (ii) biological barriers; (iii) inter-organ communication networks; and (iv) physiological protein trafficking and clearance. The following sections summarize the established biological mechanisms underlying these pillars and discuss their potential contribution to the dynamic homeostatic processes proposed by the GBHC.

**FIGURE 2:**
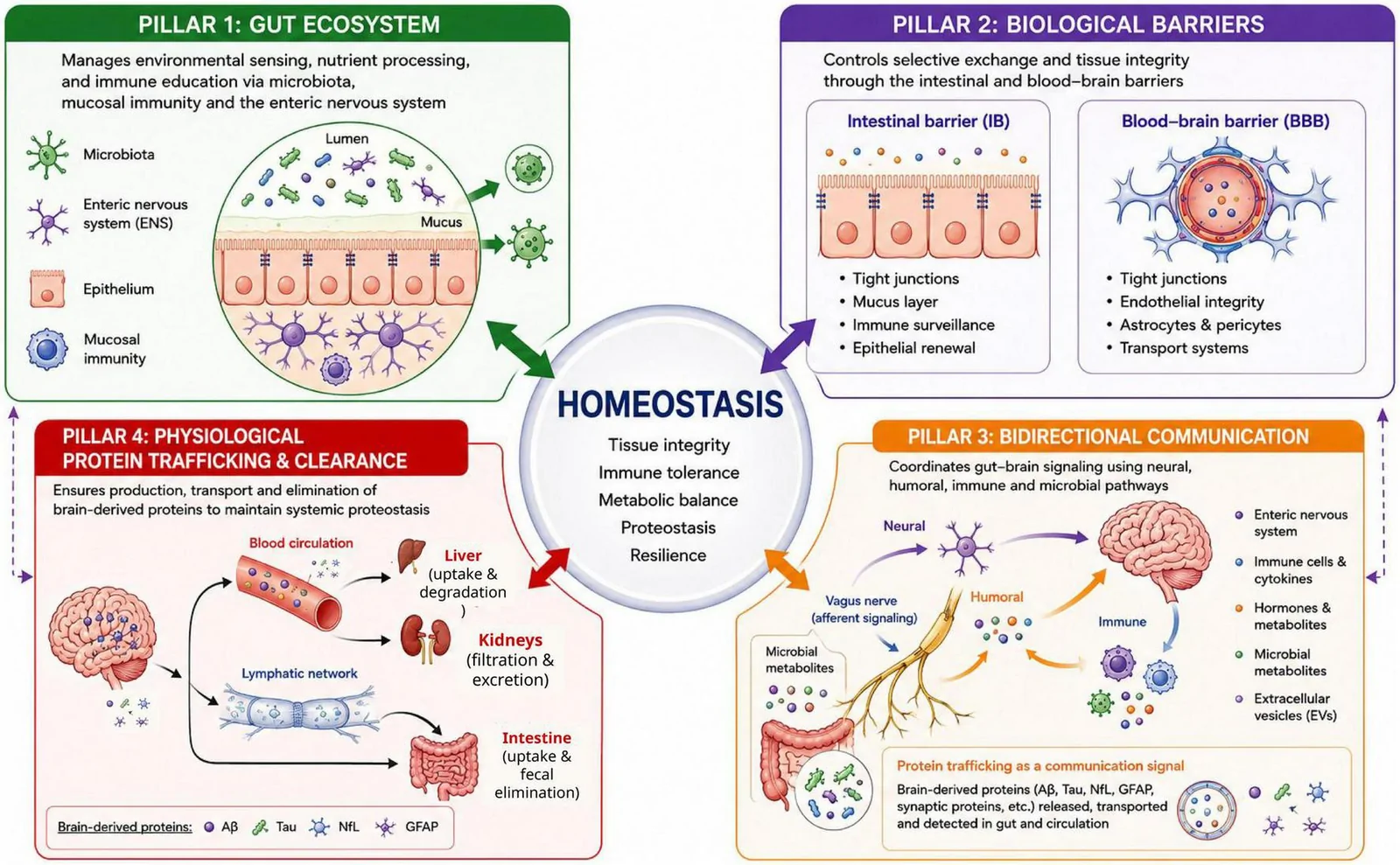
The four functional pillars of the GBHC. The GBHC is organized around four interconnected functional pillars that collectively preserve systemic and cerebral homeostasis. (1) Gut ecosystem. The intestinal ecosystem, comprising the microbiota, intestinal epithelium, ENS and mucosal immune system, forms the primary host-environment interface and supports nutrient processing, immune education and metabolic homeostasis. (2) Biological barriers. The IB and BBB function as coordinated interfaces that regulate selective molecular exchange, preserve tissue integrity and coordinate immune surveillance between the gut, the circulation and the CNS. (3) Bidirectional communication networks. Neural, endocrine, immune, metabolic and EV-mediated pathways continuously exchange information between the gut, peripheral organs and the brain, enabling adaptive responses to physiological and environmental challenges. (4) Physiological protein trafficking and clearance. This pillar encompasses communication and coordinated clearance processes involving the gut, liver, kidneys, and brain, including intestinal uptake and fecal elimination, hepatic uptake and degradation, renal filtration and excretion, and their interactions with the intestinal barrier and the blood-brain barrier/neurovascular unit. Brain-derived proteins, including Aβ, tau, NfL, GFAP and synaptic proteins, may be transported and eliminated through complementary clearance pathways involving the glymphatic and meningeal lymphatic systems, BBB transport, the liver, kidneys and gastrointestinal tract. The extent to which these pathways contribute to systemic proteostasis and fecal protein detection remains to be established. Together, these four functional pillars coordinate tissue integrity, immune tolerance, metabolic balance, proteostasis and resilience. Progressive dysfunction of one or several pillars disrupts this homeostatic continuum, promoting the transition from physiological aging toward AD.

### 2.1 The gut ecosystem

The adult gastrointestinal tract (GIT) harbors a dense and metabolically active microbial ecosystem, comprising trillions of bacteria, archaea, fungi and viruses, whose collective gene repertoire and metabolic potential greatly exceed those encoded by the human genome. Its physiological state, commonly referred to as eubiosis, is generally understood as a relatively stable and functionally balanced host-microbe ecosystem rather than a fixed microbial composition. It involves context-dependent features such as community stability, functional capacity, functional redundancy and effective host-microbe interactions (Iebba et al., 2016). Consequently, host health may depend more strongly on the preservation of microbial functions and host-microbe interactions than on the presence or abundance of individual taxa alone.

Microbial metabolism provides several signals relevant to host homeostasis. Fermentation of dietary fibers yields SCFAs, namely acetate, propionate and butyrate, which fuel intestinal epithelial cells (IEC) while promoting epithelial renewal, immune tolerance and intestinal barrier (IB) integrity (Sun et al., 2020; Williams et al., 2024). Beyond its local intestinal effects, butyrate may influence microglial function through epigenetic mechanisms, including histone deacetylase inhibition, and through SCFA receptor signaling. However, the relevance and relative contribution of these mechanisms to human neuroimmune homeostasis remain to be established (Fock and Parnova, 2023). Microbial metabolism also generates vitamins, including vitamin K and several B vitamins, neuroactive compounds, secondary bile acids and tryptophan-derived indoles that influence epithelial, immune, endocrine and metabolic functions (Tarracchini et al., 2025). Resident microorganisms further confer colonization resistance and maintenance of mucus, tight junctions and Paneth cell antimicrobial peptides, limiting microbial translocation into the systemic circulation (Iacob et al., 2018). The gut microbiota may therefore contribute to physiological homeostasis through its metabolic flexibility, functional redundancy and interactions with host tissues.

The ENS is an intrinsic neural network that integrates microbial, epithelial and immune signals to regulate gut motility, secretion and mucosal homeostasis. Through vagal and spinal pathways, it also contributes to bidirectional communication with the CNS (Furness, 2012).

From a GBHC perspective, the relevance of the gut microbiota therefore does not reside in a single microbiota-brain pathway or in the assumption that dysbiosis directly causes AD. Its contribution to the GBHC may instead relate to the capacity of the host ecosystem to maintain and adapt systemic homeostasis in response to nutritional, metabolic, immune and environmental perturbations.

### 2.2 Biological barriers: dynamic interfaces of the GBHC

#### 2.2.1 The intestinal barrier (IB): an interface for systemic homeostasis

The IB constitutes the largest interface between the host and the external environment, covering an estimated 30–40 m^2^ continuously exposed to dietary antigens, microorganisms and environmental compounds. Far from a passive wall, the IB is a dynamic multicellular system that regulates nutrient absorption while limiting uncontrolled exposure of the internal environment to microorganisms, dietary antigens and potentially harmful compounds. Its integrity depends on the coordinated activity of epithelial cells, mucus, antimicrobial mechanisms, cell-cell junctions, extracellular matrix interactions and mucosal immune populations.

#### 2.2.2 Structural organization

The IB consists of a single layer of polarized epithelial cells organized into villi and crypts of Lieberkühn, forming the crypto-villus axis (Sancho et al., 2003). Villi constitute the principal sites of nutrient absorption and harbor a dense network of blood capillaries and central lymphatic lacteals that ensure the systemic distribution of nutrients, lipids and immune mediators. In contrast, the crypts form specialized regenerative niches where epithelial renewal is continuously sustained. This remarkable regenerative capacity relies on intestinal stem cells located at the base of the crypts. These cells generate all epithelial lineages through a tightly regulated differentiation program, allowing complete epithelial renewal every 3–5 days (Cheng and Leblond, 1974; Sato et al., 2009). This rapid and tightly regulated renewal is essential for maintaining intestinal barrier integrity and, consequently, for preserving local and systemic homeostasis.

#### 2.2.3 Cellular specialization

The IB is composed of several highly specialized cell populations that collectively ensure the diverse physiological functions of the barrier. Enterocytes, the predominant epithelial cell type, mediate nutrient absorption through a broad repertoire of transporters regulating the uptake of ions, water, carbohydrates, amino acids and lipids (Snoeck et al., 2005; Miron and Cristea, 2012). Goblet cells secrete the gel-forming mucin MUC2, which creates a protective mucus layer that separates luminal microorganisms from the epithelium and provides a niche for commensal bacteria (Specian and Oliver, 1991). Located at the base of the crypts, Paneth cells release antimicrobial peptides, including α-defensins, lysozymes and REGIII proteins, shaping microbial composition and protecting the stem cell niche (Wallaeys et al., 2023). Although representing less than 1% of epithelial cells, enteroendocrine cells constitute major sensory transducers that detect luminal nutrients and microbial metabolites and respond through the secretion of hormones such as glucagon-like peptide-1 (GLP-1), peptide YY (PYY) and serotonin (Latorre et al., 2016), linking the lumen to systemic metabolism and to the ENS. Together, these specialized cell populations form a functionally integrated epithelial network in which sensing, nutrient absorption, microbial control, tissue protection and regeneration are continuously coordinated. This cellular specialization is therefore essential not only for maintaining intestinal barrier homeostasis, but also for enabling the epithelium to adapt dynamically to changes in the luminal environment.

#### 2.2.4 Cell adhesion and extracellular matrix (ECM)

Epithelial integrity relies on complementary cell–cell and cell–matrix interactions. Tight junctions, composed primarily of claudins, occludin and zonula occludens-1 (ZO-1), regulate paracellular permeability and establish the selective barrier separating the intestinal lumen from the internal environment (Farquhar and Palade, 1963; Tsukita et al., 2001). Together with adherens junctions and desmosomes, they preserve epithelial polarity, mechanical stability and tissue cohesion. In parallel, integrin-mediated interactions with the basement membrane provide structural anchorage while transmitting biochemical and mechanical signals that regulate proliferation, differentiation, migration and tissue repair (Chartier et al., 2011). Together, these interconnected mechanisms not only maintain epithelial integrity under physiological conditions but also enable the barrier to dynamically respond to mechanical, microbial and inflammatory challenges and to restore tissue organization following injury. This capacity for continuous structural maintenance and repair is therefore an essential component of intestinal homeostasis and adaptive capacity.

#### 2.2.5 Mucosal immunity

The gut-associated lymphoid tissue (GALT), including Peyer’s patches, isolated lymphoid follicles and the lamina propria, samples luminal antigens while balancing immunity and tolerance (Jung et al., 2010). Dendritic cells capture luminal antigens either directly (Rescigno et al., 2001) or through specialized microfold (M) cells overlying Peyer’s patches (Kraehenbuhl and Neutra, 2000), promoting adaptive immune responses and oral tolerance toward dietary antigens and commensal microorganisms. Resident macrophages contribute to tissue homeostasis by clearing apoptotic cells and microbial debris while limiting excessive immune activation (Meng et al., 2024). Secretory IgA, produced by plasma cells, further reinforces mucosal protection by coating luminal microorganisms without triggering inflammation (Pietrzak et al., 2020). Because immune cells also respond to neurotransmitters, neuropeptides and microbial metabolites, immune regulation is tightly interconnected with the ENS, thereby contributing to the organization of the IB as a neuroimmune ecosystem rather than a simple epithelial interface.

#### 2.2.6 Enteric nervous system

The ENS, often referred to as the “second brain”, comprises enteric neurons and enteric glial cells (EGCs) organized within the myenteric and submucosal plexuses (Furness, 2007; Benarroch, 2007). Through intrinsic neural circuits and bidirectional communication with vagal and spinal pathways, enteric neurons regulate intestinal motility, secretion, blood flow and sensory signaling while integrating luminal, microbial and immune-derived information (Furness, 2012). Beyond their classical supportive functions, EGCs have emerged as key regulators of epithelial integrity. They interact closely with epithelial cells, immune populations, the ECM and the intestinal microbiota to promote epithelial renewal, maintain tight junction organization and coordinate tissue repair (Neunlist et al., 2003, 2014). EGCs also release trophic factors, cytokines and gliotransmitters that orchestrate neuroimmune communication and modulate local inflammatory responses. Their dysfunction has been associated with barrier disruption and chronic intestinal inflammation, highlighting the essential role of the ENS in maintaining intestinal resilience (Yu, 2014).

Taken together, the microbiota, intestinal epithelium, mucosal immune system and ENS form an integrated physiological ecosystem in which sensing, protection, metabolism, regeneration and neural coordination are continuously coupled. Their interactions provide the functional basis for maintaining intestinal homeostasis and adapting to persistent changes in the internal and external environment. Within the GBHC, this coordinated gut ecosystem constitutes the first functional pillar, providing a dynamic interface through which local intestinal processes can influence, and respond to, systemic physiology.

### 2.3 The blood–brain barrier: a dynamic neurovascular interface

The BBB constitutes the principal interface between the systemic circulation and the CNS. It is recognized as a dynamic neurovascular interface regulating molecular exchange, immune surveillance and metabolic homeostasis. Within the GBHC, the BBB represents an important homeostatic interface between the peripheral circulation and the cerebral microenvironment, integrating systemic signals while preserving the specialized neuronal environment.

#### 2.3.1 Structural organization and selective permeability

The BBB covers a surface area of 12–20 m^2^ and is formed by specialized endothelial cells lining cerebral microvessels. Unlike peripheral endothelial cells, they are connected by exceptionally complex tight junctions that strongly restrict paracellular diffusion, thereby establishing a highly selective permeability barrier (Liu et al., 2012). This endothelial layer is supported by a continuous basement membrane and closely associated with pericytes, astrocytic end-feet, vascular smooth muscle cells and neural cells, which together contribute to the cellular and extracellular organization of the neurovascular unit (NVU; McConnell and Mishra, 2022). Resident immune cells, including microglia, are closely associated with the NVU and interact dynamically with its cellular components, contributing to immune surveillance, vascular homeostasis and tissue responses. As an integrated functional unit, the NVU regulates cerebral blood flow, nutrient delivery, waste elimination and immune surveillance. This structural organization allows the BBB to combine physical protection with continuous physiological regulation, thereby maintaining the stability of the cerebral microenvironment despite fluctuations in the systemic circulation.

#### 2.3.2 Cellular specialization

Brain endothelial cells regulate the selective transport of nutrients and metabolites while restricting the uncontrolled entry of circulating molecules. Pericytes contribute to vascular stability, angiogenesis and endothelial permeability (Roedel et al., 2025). Astrocytic end-feet contribute to BBB maturation and maintenance through trophic support, metabolic coupling and regulation of the perivascular environment (Schiera et al., 2024). Astrocytes regulate blood flow, ionic balance, neurotransmitter recycling and water homeostasis (Weber and Barros, 2015; Zhou et al., 2022; Mishra et al., 2024). These cellular populations collectively support the functional integrity of the NVU, which contributes to the regulation of cerebral blood flow, brain metabolic homeostasis and blood–brain barrier integrity (He and Sun, 2025). Microglia, although not considered classical structural components of the NVU, interact closely with the neurovascular unit and contribute to immune surveillance, synaptic maintenance and tissue repair.

Blood-brain barrier selectivity relies on tight junctions, in which claudin-5, occludin and ZO-1 restrict paracellular diffusion between adjacent endothelial cells, acting together with adherens junctions and the endothelial cytoskeleton. As in the IB, integrin-mediated interactions with the basement membrane transmit bidirectional signals governing endothelial polarity, vascular stability, angiogenesis and repair (Yao, 2019). These coordinated cell-cell junctions and cell-ECM interactions support BBB integrity and allow the neurovascular interface to adjust to physiological demands and respond to perturbation (Daneman and Prat, 2015).

#### 2.3.3 Integrative function

Rather than acting as a passive barrier, the BBB continuously senses and regulates the exchange of nutrients, microbial metabolites, hormones, cytokines, and EVs, allowing the brain to adapt its metabolic and immune responses to systemic conditions (Alkayed and Cipolla, 2023). Conversely, the BBB contributes to the efflux of metabolic waste products and selected soluble brain-derived molecules, including Aβ and, potentially, specific tau species, toward the circulation, thereby contributing to cerebral proteostasis and linking brain homeostasis to peripheral physiology (Ronaldson and Davis, 2022).

From a GBHC perspective, this bidirectional regulation provides a functional link between peripheral and cerebral homeostasis: systemic signals can be sensed and accommodated at the neurovascular interface while brain-derived signals and products can influence peripheral compartments.

#### 2.3.4 Coupling between biological barriers

The IB and the BBB function as coordinated interfaces. Experimental studies indicate that microbial metabolites, cytokines, endocrine mediators and EV-associated signals originating from the gut can influence BBB maturation, permeability and neurovascular function, whereas CNS activity modulates intestinal physiology through autonomic, neuroendocrine and immune pathways (Braniste et al., 2014; Cryan et al., 2019). Alterations in one barrier may be associated with functional changes in the other, creating a dynamic network of reciprocal regulation in which intestinal and cerebral homeostasis are coupled. Within the GBHC, the two barriers are consequently considered interconnected components of a broader homeostatic continuum rather than independent anatomical compartments.

### 2.4 Communication networks: connecting the pillars of the GBHC

Once the gut ecosystem and biological barriers are considered as functional interfaces, homeostasis depends on the continuous exchange of information between interconnected tissues. Neural, immune, metabolic and endocrine signals, together with EV-mediated communication, provide complementary mechanisms through which local physiological states can influence distant organs (Figure 3).

**FIGURE 3:**
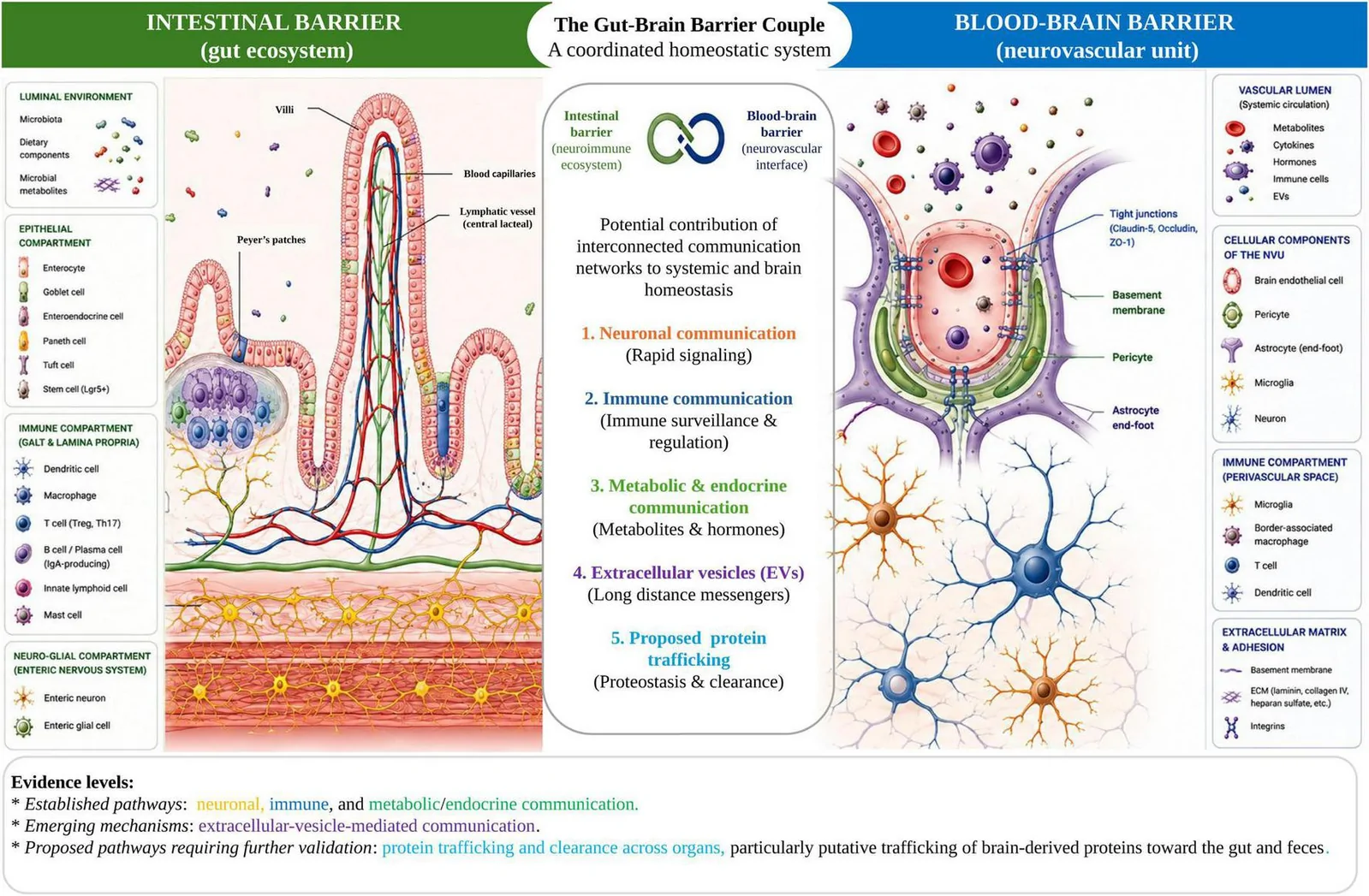
Functional communication networks within the GBHC. The IB (left) and the BBB (right) constitute the two principal homeostatic interfaces linking the gut ecosystem to the CNS. The IB comprises the epithelial layer, villus–crypt axis, blood and lymphatic vessels, GALT, and the ENS, whereas the BBB is organized around the neurovascular unit (NVU), including endothelial cells, pericytes, astrocytic end-feet, immune cells and extracellular matrix. Functional integration between these interfaces relies on five complementary communication networks: (1) neural communication, (2) immune communication, (3) metabolic & endocrine communication, (4) extracellular vesicle-mediated communication, and (5) physiological protein trafficking and clearance. Together, these interconnected pathways coordinate bidirectional communication between the gut, peripheral organs and the brain, enabling adaptive responses to physiological and environmental challenges while preserving tissue homeostasis, systemic proteostasis and organismal resilience.

#### 2.4.1 Neural communication

Neural communication represents the fastest communication network within the GBHC, allowing continuous integration of environmental, metabolic and immune information between the gut ecosystem and the CNS. Rather than operating as independent afferent and efferent pathways, the ENS interacts closely with the autonomic nervous system to form an integrated bidirectional regulatory network that dynamically coordinates intestinal physiology with cerebral function. Descending brain-to-gut pathways regulate virtually every aspect of GI physiology. Parasympathetic vagal efferents and sympathetic fibers modulate intestinal motility, epithelial secretion, mucus production, blood flow and permeability while influencing enteric neurons, EGCs and mucosal immune populations (Duan et al., 2021). In parallel, activation of the HPA axis links stress to intestinal physiology through glucocorticoid and corticotropin-releasing factor signaling, thereby modulating barrier integrity, immune responses and microbial ecology (Ducarouge et al., 2017).

Ascending gut-to-brain pathways continuously relay information from the intestinal environment to the CNS. Luminal nutrients, microbial metabolites and immune mediators are detected by specialized epithelial and neuronal sensors and rapidly transmitted through vagal and spinal afferents. A major advance has been the identification of neuropod-like processes in specialized enteroendocrine cells, which can form direct synaptic contacts with vagal neurons. These connections can transmit selected luminal information on a rapid timescale through glutamatergic signaling, providing the brain with an immediate representation of aspects of the intestinal environment (Kaelberer et al., 2020).

#### 2.4.2 Immune communication

Immune communication integrates local mucosal immunity with systemic and central immune surveillance. Following antigen sampling, intestinal dendritic cells can migrate to mesenteric lymph nodes, where they contribute to the priming and differentiation of naïve T cells (Jang et al., 2006; Cerovic et al., 2013). Cytokines and chemokines produced within the intestinal mucosa can act locally or enter the circulation, where they may regulate leukocyte trafficking, endothelial activation and barrier permeability across multiple tissues (Zimmerman et al., 2008; Muller, 2014). Under physiological conditions, this controlled inflammatory tone contributes to tissue repair, vascular homeostasis and immune surveillance without triggering chronic inflammation.

Within the CNS, microglia, border-associated macrophages and perivascular immune cells continuously monitor the cerebral microenvironment. Although the BBB restricts uncontrolled immune cell entry, circulating cytokines and tightly regulated immune-cell trafficking allow peripheral immune status to influence central immune surveillance while preserving neural homeostasis (Ruiz-Rodríguez et al., 2026). Reciprocally, neuroendocrine pathways provide a direct feedback loop from the brain to the gut. In some bacterial species, including certain enteric pathogens, catecholamines such as adrenaline and noradrenaline can be sensed through receptors such as QseC, influencing bacterial growth, motility and virulence-associated gene expression (Hughes and Sperandio, 2008). Glucocorticoid signaling and mast-cell activation can affect epithelial barrier properties, whereas neuroendocrine and inflammatory signals may also modify mucus production and composition (Rubio and Huang, 1992; Demaude, 2006). Thus, immune communication operates as a distributed regulatory network in which local immune states can be sensed, amplified, constrained or resolved across anatomical compartments. This reciprocal regulation allows immune responses to adapt to microbial and environmental challenges while limiting persistent inflammation, thereby contributing to the maintenance of systemic homeostasis.

#### 2.4.3 Metabolic and endocrine communication

Microbial metabolites constitute major biochemical messengers connecting the gut ecosystem with peripheral organs and the brain. SCFAs regulate glycaemia and lipid metabolism (Jones, 2016), stimulate goblet-cell mucus secretion and reinforce epithelial tight junctions (Burger-van Paassen et al., 2009; Peng et al., 2009). In experimental models, SCFAs have been reported to influence microglial maturation and function, BBB integrity and neuronal metabolism through receptor-mediated and epigenetic mechanisms (Yassin et al., 2025). The extent to which these effects contribute to human brain homeostasis remains to be established.

Beyond SCFAs, other microbiota-derived metabolites can also modulate gut-brain communication. A recent experimental study reported that age-associated expansion of *Parabacteroides goldsteinii* was associated with the production of medium-chain fatty acids, activation of GPR84 signaling in peripheral myeloid cells and inflammatory suppression of vagal afferent activity (Cox et al., 2026). The authors further linked these changes to reduced hippocampal activation and impaired memory encoding. These findings provide experimental evidence for a possible microbiota-immune-vagal-brain pathway, but their relevance to human aging and AD remains to be determined.

Bile acids, diversified by microbial metabolism, activate host receptors including farnesoid X receptor and Takeda G protein-coupled receptor 5, thereby regulating lipid and glucose metabolism, barrier function, immune responses and neuroinflammation (Pathak et al., 2017; Song et al., 2025; Zangerolamo et al., 2025). Tryptophan metabolism provides another major communication pathway. Host kynurenine and serotonin pathways, together with microbial production of aryl hydrocarbon receptor (AhR)-activating indoles, influence epithelial renewal, mucosal immunity and, through context-dependent mechanisms, astrocyte, microglial and neuronal functions (Ye et al., 2022; Holeček, 2026). These observations illustrate how microbiota-derived metabolites can act as systemic signals rather than local microbial products, simultaneously influencing epithelial integrity, immune tone, vascular function and neural activity. Their effects therefore provide a mechanistic link between the metabolic state of the gut ecosystem and the physiological state of distant organs.

This metabolic network is reinforced by enteroendocrine and endocrine signaling. GLP-1 and PYY, released by intestinal L cells in response to nutrients and microbial metabolites, regulate satiety, insulin secretion, GI motility and vagal activity (Gribble and Reimann, 2019). GLP-1 signaling has been reported to exert neuroprotective and anti-inflammatory effects and to influence BBB integrity, particularly in experimental models (Wong et al., 2024). Insulin coordinates glucose utilization and influences neuronal survival, synaptic plasticity and cerebral energetics (Arnold et al., 2018), whereas cortisol couples stress responses to metabolic adaptation by regulating permeability, immune function and glucose metabolism (De Punder and Pruimboom, 2015).

Together, neural, immune, metabolic and endocrine pathways form complementary communication networks linking intestinal, systemic and cerebral physiology. Within the GBHC, this communication layer connects the gut ecosystem, biological barriers and distant organs, allowing local changes to be translated into coordinated systemic responses. Persistent dysregulation across several of these pathways may progressively reduce the capacity to adapt to perturbations and restore homeostasis. EVs represent a particularly versatile component of this network because they can carry multiple classes of biological information within single structures.

### 2.5 Extracellular vesicles: emerging biological carriers in the GBHC

Extracellular vesicles have recently emerged as major mediators of intercellular communication, complementing the classical neural, endocrine, immune and metabolic pathways that underpin the GBHC. Secreted by virtually all living cells, including bacteria, EVs are nanosized lipid bilayer-enclosed particles that transport proteins, lipids, metabolites and nucleic acids between neighboring and distant cells (Kalluri and LeBleu, 2020; Benameur et al., 2025). Unlike many soluble mediators, EVs can simultaneously transport multiple classes of molecular cargo, whose composition reflects the physiological state of the producing cell. Their lipid bilayer protects this cargo from degradation in biological fluids and enables selective interactions with recipient cells. Some EV populations can cross or influence biological barriers, including the IB and, under specific physiological or pathological conditions, the BBB, thereby enabling complex molecular information to be conveyed over long distances (Chen et al., 2025; Figueroa-Hall et al., 2025; Hunter et al., 2025). However, the extent to which EV-mediated communication contributes to physiological resilience and its alteration during AD remain to be established.

According to their cellular origin, EVs can be broadly classified into bacterial EVs (bEVs) and host-derived EVs (hEVs). Although differing in their biogenesis and molecular composition, both populations participate in continuous bidirectional communication between the intestinal microbiota, epithelial and immune cells, peripheral organs and the CNS. Their cargo varied according to microbial composition, diet, aging, inflammation and metabolic status, providing a potential mechanism through which changes in local physiological states may be transmitted to distant tissues.

#### 2.5.1 Bacterial extracellular vesicles

Bacterial EVs are continuously released by the gut microbiota and represent a major mechanism through which microorganisms communicate with their host beyond direct bacterial colonization. They include outer membrane vesicles from Gram-negative bacteria and membrane vesicles from Gram-positive species, often reported within a broad nanoscale range, although their size distribution varies according to bacterial species and isolation methods (Benameur et al., 2025). Their cargo reflects the molecular composition of the parental bacterium and contains pathogen-associated molecular patterns (PAMPs), including lipopolysaccharides (LPS), peptidoglycan fragments and lipoproteins, together with nucleic acids, metabolites and bacterial enzymes. Within the intestinal ecosystem, bEVs contribute to bacterial competition, biofilm organization and horizontal gene transfer while interacting with epithelial cells, goblet cells, enteroendocrine cells and resident immune populations. Through activation of pattern-recognition receptors (PRRs), particularly Toll-like receptors (TLRs) and NOD-like receptors, they shape innate and adaptive immune responses (Martínez-Ruiz et al., 2023; Sun et al., 2023). Depending on their microbial origin, bEVs either promote immune tolerance and epithelial homeostasis or trigger inflammatory signaling associated with dysbiosis. Importantly, bEVs may allow microbial signals to reach distant organs without bacterial translocation. Experimental studies indicate that some bEVs can be internalized by intestinal epithelial cells and cross the intestinal interface under specific conditions (Stentz et al., 2018; Turner et al., 2018), enter the circulation and disseminate to peripheral tissues, including the liver (Zhou et al., 2025), while also reaching the CNS through the BBB (Han et al., 2019). Our previous work demonstrated that circulating bEVs are detectable in healthy blood donors despite the absence of measurable barrier disruption, where they preferentially interact with circulating monocytes (Schaack et al., 2022). Moreover, orally administered fluorescent bEVs rapidly disseminate to multiple organs and persist for up to 3 days in mice (Schaack et al., 2024). In mice, orally administered vesicles from *Paenalcaligenes hominis* were reported to reach the hippocampus through a process partly dependent on vagal signaling, as vagotomy reduced their hippocampal accumulation (Lee et al., 2020). In experimental models, the biological effects of bEVs depend strongly on their bacterial origin and molecular cargo. Vesicles from selected commensal bacteria have been reported to support barrier integrity or immune tolerance, whereas pathogen-derived vesicles can promote inflammatory responses through the delivery of components such as LPS (Aoki-Yoshida et al., 2017; Sun et al., 2023; Zhao et al., 2019; Gong et al., 2022; Yoshida et al., 2023). These findings indicate that bEVs can disseminate beyond the intestinal compartment and influence peripheral and cerebral tissues, providing a potential mechanism for microbiota–host and gut–brain communication.

#### 2.5.2 Host extracellular vesicles

Most host cell types release EVs under physiological and pathological conditions, including small and large EVs, whose molecular cargo reflects aging, diet, physical activity, metabolic status, inflammation and microbial signals (Van Niel et al., 2018; Mathieu et al., 2021; Welsh et al., 2024). Within the GIT, EVs released by epithelial and immune cell populations have been implicated in epithelial maintenance, barrier regulation and mucosal immune communication (Bui et al., 2018). Conversely, epithelial stress, infection and chronic inflammation profoundly remodel EV biogenesis and cargo composition, promoting dissemination of pro-inflammatory mediators and regulatory nucleic acids that amplify intestinal dysfunction (Buzas, 2023; Sun et al., 2023). Beyond the intestine, hEVs establish an extensive communication network linking metabolic and vascular organs. Hepatocyte-derived EVs regulate lipid and glucose metabolism, adipocyte-derived vesicles influence systemic energy homeostasis and insulin sensitivity, whereas endothelial- and platelet-derived EVs contribute to vascular homeostasis, angiogenesis and hemostasis while coordinating inflammatory and metabolic responses throughout the circulation (Akbar et al., 2019).

Within the CNS, neurons, astrocytes, microglia and endothelial cells all participate in EV-mediated communication. Neuronal EVs regulate synaptic plasticity and connectivity (Solana-Balaguer et al., 2023), astrocytic EVs provide metabolic support, microglial EVs participate in synaptic remodeling and inflammatory regulation, whereas endothelial EVs contribute to BBB integrity (Gharbi et al., 2020; Li et al., 2024; Wies Mancini et al., 2024). HEVs should not be considered solely as static biomarkers. Their cargo can be remodeled in response to physiological and environmental cues, while the vesicles themselves may also act as biological effectors. This dual role makes them potential indicators of tissue state and possible mediators of inter-organ adaptation. Whether these properties can be exploited to quantify changes in systemic physiological resilience remains an important question for future research.

Collectively, bEVs and hEVs constitute a complementary layer of inter-organ communication that interacts with neural, immune, endocrine and metabolic pathways. Their capacity to transport complex, context-dependent cargo across tissues and, under specific conditions, biological barriers provides a plausible mechanism through which local physiological states may influence distant organs. Within the GBHC, EVs may therefore represent an important communication layer linking the gut ecosystem, biological barriers and peripheral and cerebral compartments. Their precise contribution to homeostatic adaptation, loss of resilience and AD progression remains to be experimentally established.

### 2.6 Physiological protein trafficking and clearance within the Gut–Brain Homeostatic Continuum

A central proposition of the GBHC is the concept of a Gut Protein Clearance Continuum, which extends the classical view of cerebral proteostasis by considering brain-derived (BD) proteins within an interconnected central-to-peripheral clearance network. In this framework, proteins produced or released within the CNS may be transferred to peripheral compartments through multiple complementary pathways, with the GIT representing a potential downstream interface of systemic protein handling. While several steps of this network are well established, the extent to which BD-proteins ultimately reach the intestinal lumen under physiological conditions remains to be determined.

Before their aberrant misfolding and aggregation driving the hallmark pathology of AD, both Aβ and tau are produced continuously throughout life and fulfill important biological functions: soluble Aβ has been implicated to synaptic plasticity, neuronal excitability and antimicrobial defense, whereas tau regulates microtubule dynamics, axonal transport and cytoskeletal stability (Kent et al., 2020). Brain proteostasis relies on a complex network of complementary pathways operating at both the intracellular and systemic levels (Figure 4). Intracellular tau is degraded by the ubiquitin-proteasome and autophagy-lysosomal systems (Wang et al., 2025), whereas extracellular Aβ is eliminated through microglial and astrocytic phagocytosis (Ries and Sastre, 2016), enzymatic degradation by neprilysin and insulin-degrading enzyme (Yasojima et al., 2001; Farris et al., 2003), glymphatic and meningeal lymphatic drainage (Formolo et al., 2023), receptor-mediated efflux across the BBB through low-density lipoprotein receptor-related protein-1 (LRP1) and P-glycoprotein (P-gp; Shibata et al., 2000; Chiu et al., 2015), and peripheral degradation by the liver and kidneys (Ghiso et al., 1997; Tamaki et al., 2006; Tian et al., 2021; Cheng et al., 2023). Together, these complementary mechanisms contribute to the continuous balance between protein production, processing and removal that is required to preserve cerebral proteostasis.

**FIGURE 4:**
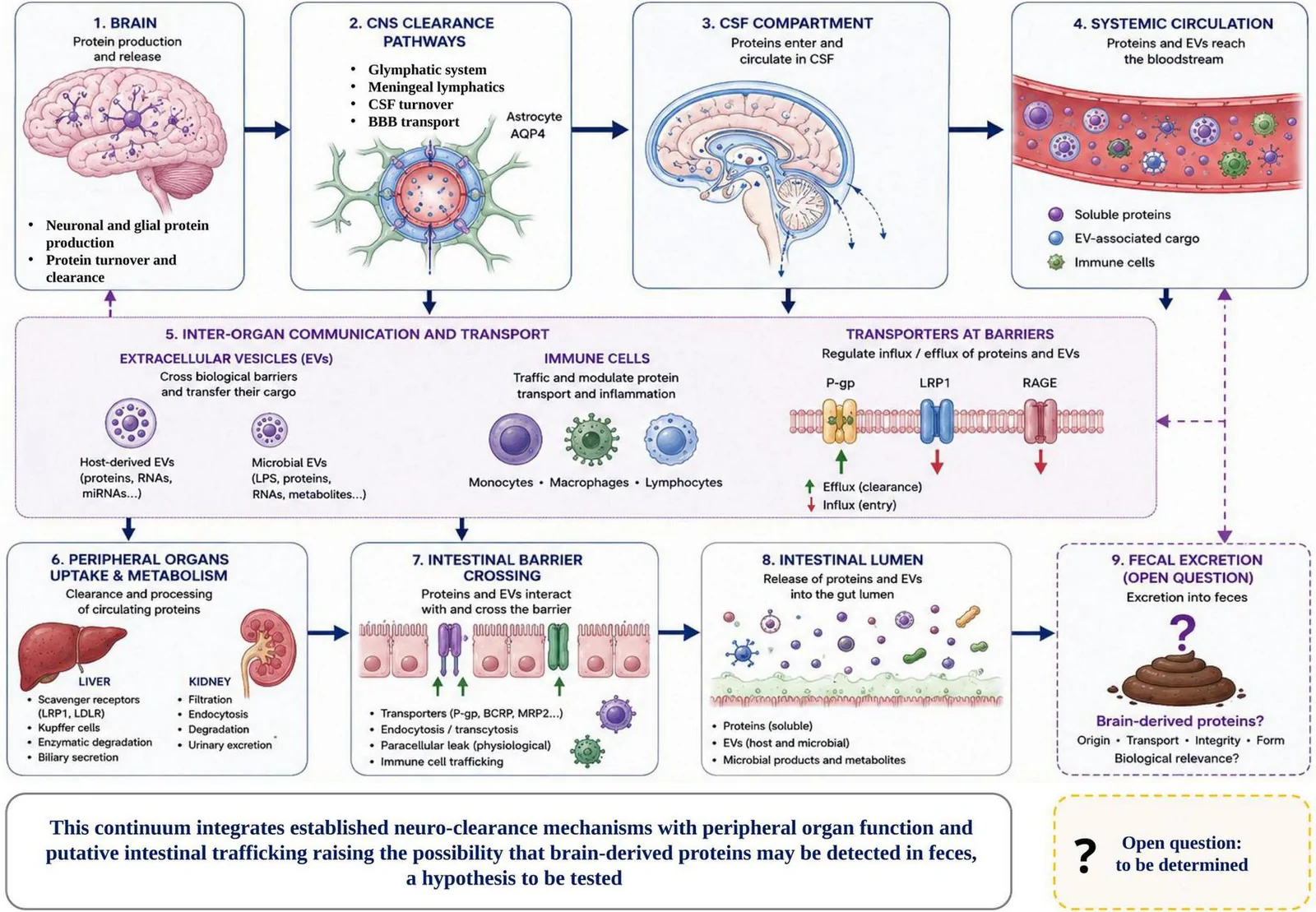
The gut protein clearance continuum within the GBHC. Brain proteins are continuously cleared through interconnected central and peripheral pathways. Within the CNS, protein elimination involves the glymphatic and meningeal lymphatic systems, CSF circulation and BBB transport, allowing brain-derived proteins and protein-associated cargo to reach the systemic circulation in soluble or EV-associated forms. In the periphery, circulating proteins and EVs undergo tissue-specific uptake, metabolism and redistribution involving peripheral organs, including the liver and kidneys, as well as immune cells and other circulating compartments. At the intestinal level, circulating proteins and EVs may interact with and cross the intestinal barrier through physiological trafficking mechanisms, potentially allowing their transfer into the gut lumen and subsequent fecal excretion. The extent to which brain-derived proteins follow this route, their molecular form and their biological origin in feces remain to be established. The question mark highlights this unresolved step and defines a major research opportunity within the GBHC framework. Together, these interconnected processes illustrate the proposed Gut Protein Clearance Continuum, linking cerebral proteostasis to peripheral clearance and raising the possibility that intestinal and fecal compartments may provide access to information on brain-derived protein trafficking.

These clearance pathways indicate that the brain is not an isolated organ. Following their release into the interstitial fluid, BD-proteins may cross the BBB through receptor-mediated transport, drain through glymphatic and meningeal lymphatic systems, enter the CSF or be released within EVs, potentially contributing to the appearance of brain-associated molecular species in peripheral circulation. Consistent with this connectivity, Aβ, tau, neurofilament light chain (NfL), glial fibrillary acidic protein (GFAP) and several synaptic proteins can be detected in CSF, blood and other peripheral biofluids (Georgakas et al., 2023). However, their presence in peripheral compartments does not by itself establish the route, timing or quantitative contribution of their transport from the CNS. Recent experimental observations further raise the possibility that AD-related protein signatures may also be detectable beyond the circulation. In APP/PS1 mice, we recently detected Aβ- and Tau-immunoreactive species in fecal extracts, with genotype-associated differences in the intensity of several Aβ and phosphorylated Tau signals, including changes detectable at an early age (Seignobos et al., 2026). These findings demonstrate the feasibility of detecting AD-related protein immunoreactivities in fecal material, but do not establish that the detected proteins are directly derived from the CNS. Their biological origin remains unresolved and may include central, intestinal, peripheral or circulating sources. The GIT may therefore represent a downstream compartment within a broader protein-handling network. The key question is whether coordinated protein trafficking, processing and clearance across the CNS, circulation, peripheral organs and GIT contribute to systemic proteostasis.

Once present in the systemic circulation, circulating Aβ can be taken up by peripheral organs, including the liver and kidneys, which contribute to its metabolism and elimination (Ghiso et al., 2004; Tamaki et al., 2006). These observations support a model in which cerebral proteostasis depends, at least in part, on coordinated interactions with peripheral clearance organs. Whether this network ultimately involves the GIT remains an important open question. The gut could potentially act as a downstream interface in which circulating proteins, protein fragments or vesicle-associated cargoes are captured, transformed, transported or eliminated. We propose the Gut Protein Clearance Continuum as a testable hypothesis rather than an established clearance pathway.

Physiological protein trafficking thus constitutes a potential inter-organ network contributing to systemic proteostasis. Within the GBHC, this organization extends the concept of brain proteostasis beyond individual organs and suggests that aging, genetic susceptibility and environmental exposures may alter interconnected clearance and communication mechanisms. How these processes change across the trajectory toward AD is explored in section “3 From homeostasis to collapse: the loss of adaptive resilience.”

## 3 From homeostasis to collapse: the loss of adaptive resilience

### 3.1 Physiological resilience: maintaining homeostasis before disease

Homeostasis is not a static equilibrium but a dynamic process of continuous adaptation. Within the GBHC, fluctuations in diet, infections, stress, aging and microbial composition constantly challenge physiological function. Yet many perturbations can be buffered, at least temporarily, by compensatory mechanisms operating across the gut, peripheral organs and the brain. This capacity to withstand perturbations while preserving essential biological functions defines physiological resilience. Within the GBHC, we propose that resilience may emerge from the coordinated activity of biological barriers, neural, immune and metabolic communication networks, EVs and physiological protein trafficking. These interconnected processes could collectively support the maintenance of systemic homeostasis and modulate the response to repeated or persistent challenges.

#### 3.1.1 Adaptive reserve and compensatory mechanisms

A fundamental question in AD is why individuals exposed to multiple and combined risk factors, including aging, dysbiosis, metabolic disorders and chronic inflammation, often remain clinically asymptomatic for decades. The concepts of brain reserve and cognitive reserve have substantially improved our understanding of why individuals tolerate considerable neuropathology before developing clinical symptoms (Stern et al., 2020). Building on the broader concept of physiological resilience, we propose the term systemic adaptive reserve to refer specifically to the hypothetical capacity of interconnected physiological systems, including the gut ecosystem, biological barriers, inter-organ communication networks, EV-mediated signaling and protein trafficking, to compensate for repeated perturbations while preserving organismal homeostasis. In this framework, systemic adaptive reserve represents a potential functional basis of the physiological resilience proposed within the GBHC. Unlike brain reserve and cognitive reserve, which primarily address the relationship between brain characteristics, neuropathology and clinical expression, systemic adaptive reserve refers to the adaptive capacity of interconnected physiological systems.

Maintaining physiological stability despite changing conditions is closely related to the established concept of allostasis, whereby stability is achieved through adaptive change rather than constancy. Repeated or persistent challenges may, however, impose cumulative physiological demands, contributing to what has been termed allostatic load. Within the GBHC, increasing allostatic load is proposed as a potential factor that could progressively challenge the compensatory capacity of interconnected systems. This does not imply that allostatic load or declining systemic adaptive reserve has been established as a causal mechanism of AD; rather, it provides a conceptual framework for considering how multiple low-grade perturbations might cumulatively affect physiological resilience. As compensatory capacity becomes constrained, perturbations that were previously tolerated may produce increasingly disproportionate functional consequences. The GBHC therefore hypothesizes that declining systemic adaptive reserve may narrow the range over which interconnected physiological systems can compensate for perturbation, thereby increasing vulnerability to pathological dysfunction. This proposed transition from compensated perturbation to maladaptation provides the conceptual basis for the tipping-point model presented in Figure 5.

**FIGURE 5:**
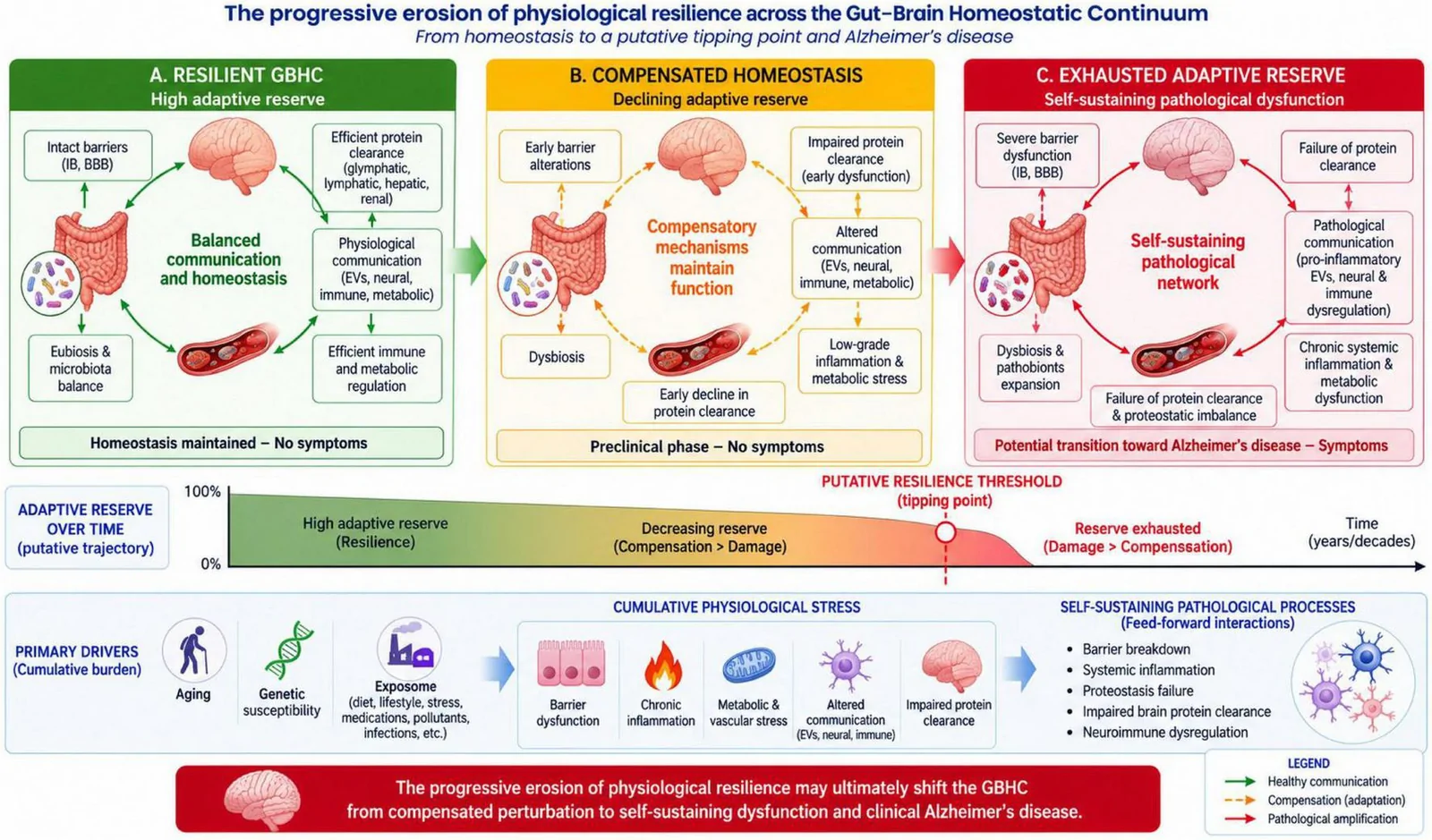
Progressive erosion of physiological resilience across the GBHC. The GBHC is proposed as a dynamic homeostatic network whose capacity to buffer physiological perturbations progressively declines with aging and cumulative exposure to genetic, environmental and lifestyle-related stressors. **(A)** Resilient GBHC: high adaptive reserve maintains barrier integrity, balanced gut microbiota, physiological inter-organ communication, immune and metabolic regulation, and efficient protein clearance, with preservation of homeostasis and absence of clinical symptoms. **(B)** Silent compensation: as cumulative physiological stress increases, early alterations in barrier function, microbiota, inter-organ communication and protein clearance emerge but remain compensated by adaptive mechanisms, allowing a prolonged preclinical phase without overt symptoms. **(C)** Exhausted adaptive reserve: when compensatory capacity falls below a critical threshold, the system reaches a tipping point beyond which pathological processes become self-sustaining. Barrier dysfunction, systemic inflammation, altered communication, proteostasis failure and impaired brain protein clearance interact through feed-forward mechanisms, ultimately contributing to progressive neurodegeneration and clinical Alzheimer’s disease. The lower panel summarizes the major drivers, cumulative physiological stressors and self-sustaining pathological processes associated with the progressive loss of adaptive reserve over time.

#### 3.1.2 The tipping point: when compensation becomes insufficient

The GBHC proposes that a progressive loss of adaptive reserve may eventually lead to a putative tipping point beyond which compensatory mechanisms become insufficient to restore homeostasis. At this stage, disturbances that were previously tolerated may propagate more readily across interconnected compartments, potentially generating self-reinforcing interactions in some contexts and increasing vulnerability to pathological dysfunction.

Within this framework, the transition toward AD is hypothesized to involve not only the accumulation of pathological changes but also a shift in the behavior of an interconnected adaptive network–from compensated perturbation toward progressively less effective compensation and self-reinforcing dysfunction. Once compensatory capacity becomes insufficient, previously reversible disturbances may become more difficult to resolve and could contribute to disease progression. This tipping-point concept remains a systems-level hypothesis and should not be interpreted as an established biological threshold in AD.

#### 3.1.3 Biological changes in AD occur long before symptoms

Alzheimer’s disease is defined biologically by evidence of Alzheimer’s disease neuropathologic change, particularly amyloid- and tau-related biomarker abnormalities, irrespective of the presence or absence of clinical symptoms (Jack et al., 2018). The GBHC extends this established disease-trajectory concept conceptually upstream by proposing that a prolonged phase of systemic physiological adaptation may coexist with accumulating molecular, cellular and systemic alterations while clinical function remains preserved.

Clinical symptoms generally emerge after a prolonged period of biological change, although the timing of clinical expression varies between individuals. From the GBHC perspective, this raises the hypothesis that progressive erosion of physiological resilience across interconnected biological systems may contribute to the transition from compensated biological change to clinically manifest disease. This hypothesis remains to be experimentally tested.

### 3.2 Drivers of resilience loss

#### 3.2.1 Aging: a major contributor to resilience loss

Aging is the strongest risk factor for sporadic AD and is accompanied by progressive alterations across multiple components of the GBHC, including the intestinal ecosystem, biological barriers, immune and vascular systems, and cerebral clearance pathways. These convergent changes may gradually reduce adaptive capacity and increase vulnerability to additional biological stressors.

Age-related alterations are also evident within the gut ecosystem. Aging is frequently associated with changes in microbial diversity, depletion of some short-chain fatty acid-producing taxa, including *Faecalibacterium, Roseburia* and other Lachnospiraceae, and enrichment of potentially pro-inflammatory taxa, particularly within the phylum Pseudomonadota. However, the direction and magnitude of these changes vary according to diet, medication use, geography, frailty and comorbidity (Claesson et al., 2012; O’Toole and Jeffery, 2015; Fan and Pedersen, 2021; Gao et al., 2026; Kim, 2026). Such ecological changes may be associated with reduced metabolic flexibility and contribute to the chronic low-grade inflammatory state known as inflammaging.

Aging also affects both biological barriers. In the intestine, epithelial senescence, impaired renewal, alterations in tight junctions and reduced mucus and antimicrobial peptide production may increase permeability and facilitate the systemic dissemination of microbial products (Turner, 2009; Allaire et al., 2018; Kayama et al., 2020). Persistent innate immune activation may further amplify this inflammatory state. Within the CNS, microglial priming, astrocyte reactivity and progressive loss of BBB integrity are accompanied by alterations in glymphatic and meningeal lymphatic drainage. Together, these changes may compromise physiological protein trafficking and cerebral proteostasis (Nikodemova et al., 2016; Palmer and Ousman, 2018; Nedergaard and Goldman, 2020; Voumvourakis et al., 2023; Bu et al., 2026). Age-related decline also affects peripheral organs involved in protein elimination, including the liver and kidneys (Mangoni and Jackson, 2004).

During early aging, functional redundancy and compensatory mechanisms may help preserve physiological function despite accumulating perturbations. As these mechanisms become progressively constrained, the range of challenges that can be effectively compensated for may narrow.

#### 3.2.2 Genetic susceptibility determines the resilience threshold

Unlike aging, which affects every individual, genetic susceptibility may modify the resilience threshold. The Apolipoprotein E (APOE) ε4 allele remains the strongest genetic risk factor for late-onset AD (Jackson et al., 2024). Beyond lipid transport and neuronal membrane maintenance, APOE regulates innate immunity, microglial activation, BBB integrity and Aβ clearance (Yamazaki et al., 2019; Martínez-Martínez et al., 2020). The ε4 isoform has distinct structural and lipid-binding properties that may alter these functions in a context-dependent manner (Nguyen et al., 2014; Ray et al., 2017). Importantly, the influence of APOE extends beyond the CNS. APOE genotype has been associated with differences in gut microbial composition and metabolite profiles in humans and experimental models (Tran et al., 2019). Other studies have reported genotype-associated differences in microbial and inflammatory features, although their consistency and functional significance remain to be established (Bressa et al., 2025). More broadly, genome-wide association studies have identified more than 75 AD susceptibility loci converging on biological pathways involved in innate immunity, membrane composition, lipid metabolism, vesicular trafficking, protein clearance and BBB integrity (Bellenguez et al., 2022; Hanbouch et al., 2022). Some of these genes are also involved in epithelial barrier function, immune responses or EV biology, suggesting that genetic susceptibility may influence several components of the physiological network represented by the GBHC rather than acting exclusively through the brain.

Consistent with this systemic view, polygenic risk score analyses have identified genetic associations between host variants influencing the abundance of several gut microbial genera and Alzheimer’s disease status, supporting a potential link between host genetic susceptibility and microbial ecology (Cammann et al., 2023). Within the GBHC framework, these observations suggest that high-risk alleles may modify resilience by reducing the adaptive capacity available to respond to aging, dysbiosis and environmental challenges.

#### 3.2.3 The exposome continuously remodels resilience

Whereas genetic susceptibility is inherited, the exposome remains modifiable throughout life. Diet, physical activity, sleep, medications, infections, psychological stress and environmental pollutants continuously influence microbial ecology, immune and metabolic responses, and biological barrier integrity.

Diet is a major long-term determinant of gut microbial composition and function. Fiber-rich diets promote microbial diversity, functional redundancy and SCFA production, whereas Western dietary patterns have been associated with microbial configurations characterized by reduced production of beneficial metabolites and increased inflammatory potential (Statovci et al., 2017; Shah et al., 2025; Ray and Shankaran, 2026). Regular physical activity has been associated with changes in microbial diversity, microbial metabolite production, vascular function and systemic inflammatory tone (Monda et al., 2017; Nicolas et al., 2024). Conversely, chronic sleep disruption impairs glymphatic clearance and increases intestinal permeability (Withrow et al., 2021). Repeated antibiotic exposure, proton pump inhibitors and non-steroidal anti-inflammatory drugs can substantially alter microbial ecology, with effects that may persist beyond treatment in some individuals (Bruno et al., 2019; Patangia et al., 2022). Obesity, type 2 diabetes, smoking, alcohol and drug consumption and environmental pollutants further increase biological stress (Durazzo et al., 2014; Chin-Chan et al., 2015; Cummings et al., 2022; Flores-Cordero et al., 2022; Chandrashekar et al., 2023).

Although individual exposures may have modest effects when considered separately, their cumulative impact may increase the allostatic load imposed on interconnected physiological systems. Across the lifespan, aging, genetic susceptibility and environmental exposures may interact to alter microbial resilience, metabolic flexibility and the capacity of homeostatic mechanisms to recover from repeated challenges. Together, these factors may create conditions that favor a transition from physiological adaptation toward pathological dysfunction.

### 3.3 Early gut ecosystem alterations as a potential marker of declining resilience

Alzheimer’s disease develops over decades before overt cognitive impairment. During this preclinical phase, subtle alterations may emerge within the gut ecosystem while systemic homeostasis remains apparently preserved. Within the GBHC framework, these changes may represent detectable manifestations of altered physiological resilience. Disruption of host–microbiota interactions could therefore accompany, and potentially contribute to, subsequent systemic and brain-related dysfunction.

Experimental studies indicate that these microbial alterations are unlikely to be merely secondary to neurodegeneration. Fecal microbiota transplantation from patients with AD induces cognitive deficits and impairs hippocampal neurogenesis in young microbiota-depleted rodents, while serum from the same patients reduces neurogenesis in human hippocampal progenitor cells, providing experimental evidence that gut-associated and circulating factors can influence neurogenesis and cognitive-related phenotypes in animal and cellular models (Grabrucker et al., 2023). Gavage with fecal samples from patients with AD, or with aging-associated bacterial species including *Paenalcaligenes hominis* and *Escherichia coli*, induces both colitis and cognitive deficits in mice (Lee et al., 2020). Conversely, microbiota from healthy donors attenuates pathology in transgenic AD models, whereas antibiotic-mediated microbiota manipulation has been reported to reduce cerebral amyloid deposition in selected transgenic AD models (Dodiya et al., 2020; Li et al., 2025). These findings support a causal contribution in animal models, which remains to be established in humans.

Clinical studies further indicate that gut microbial alterations are detectable early along the AD continuum (Figure 6 and Supplementary Table 1). Individuals with subjective cognitive decline (SCD) have been reported to exhibit alterations in gut microbial richness, diversity and taxonomic composition, although the specific patterns vary across cohorts and analytical approaches (Duan et al., 2021; Sheng et al., 2021). Shotgun metagenomic analyses have also identified differences in microbial features in cognitively unimpaired individuals with amyloid-positive biomarker profiles, suggesting that gut microbial alterations may be detectable before measurable cognitive decline (Ferreiro et al., 2023). Across SCD, mild cognitive impairment (MCI) and AD cohorts, reported changes include taxa commonly associated with SCFA production, such as *Faecalibacterium* and members of the Lachnospiraceae family, as well as taxa associated in some studies with inflammatory phenotypes, including Enterobacteriaceae and *Escherichia*/*Shigella*. However, the direction and magnitude of these associations differ according to cohort characteristics, geographical population and analytical methodology (Liu et al., 2019; Sheng et al., 2021; Verhaar et al., 2022; Wanapaisan et al., 2022; Kang et al., 2025; Xiaoyi et al., 2026). Thus, although no universal microbial signature has emerged, several studies converge on the possibility of gut ecosystem alterations before or during early cognitive impairment.

**FIGURE 6:**
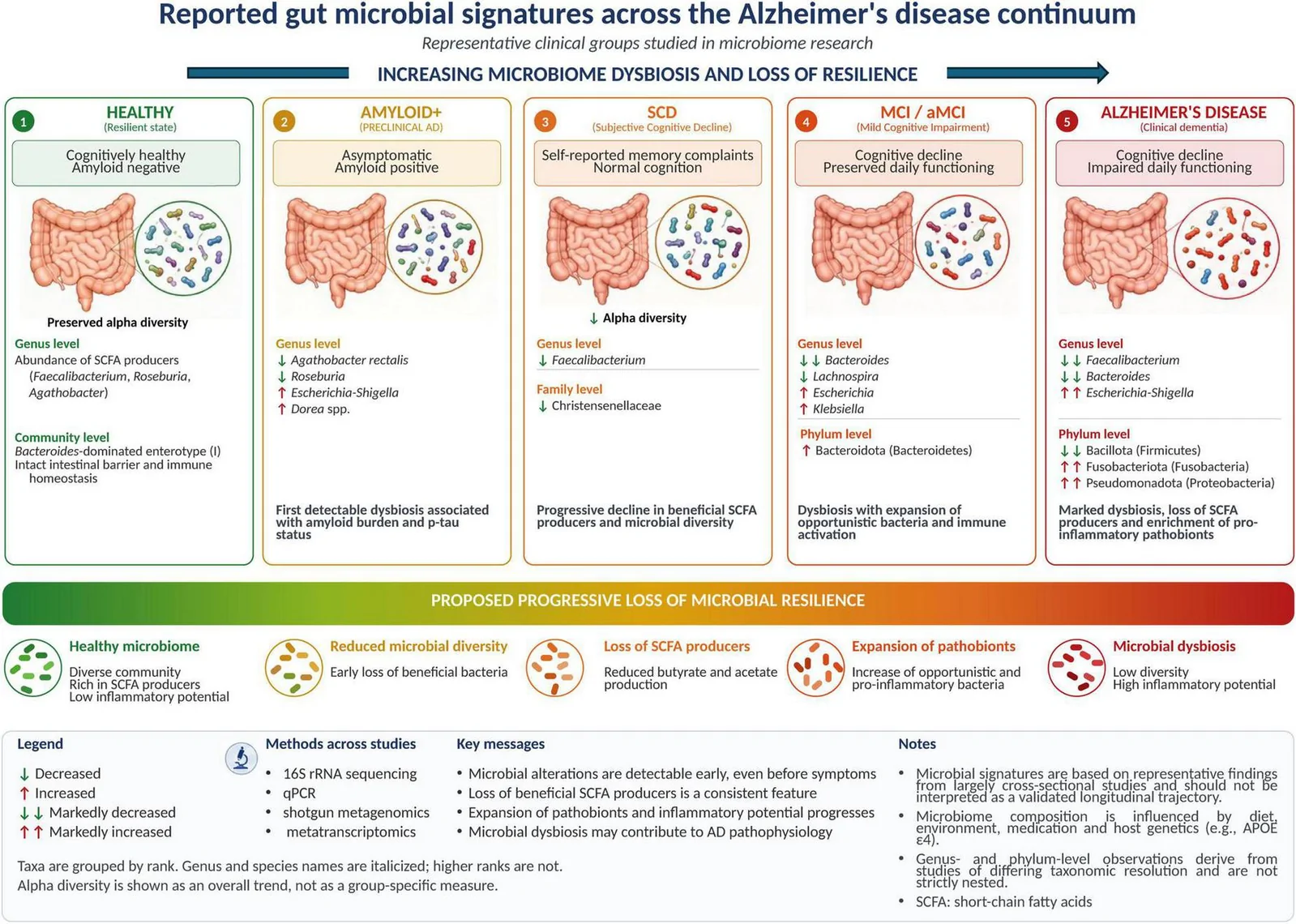
Progressive gut microbial remodeling across the AD continuum. Human studies increasingly suggest that gut microbial alterations may emerge during the preclinical stages of AD and may evolve across the clinical continuum, although reported microbial signatures vary substantially across studies. Early amyloid-positive individuals exhibit subtle reductions in butyrate-producing bacteria, including *Eubacterium rectale*, *Roseburia* spp. and members of the Lachnospiraceae family, together with enrichment of opportunistic taxa such as *Escherichia/Shigella*. These alterations become more pronounced during SCD and MCI, with progressive depletion of SCFA-producing bacteria, particularly *Faecalibacterium*, *Lachnospira* and *Bacteroides*, accompanied by expansion of Enterobacteriaceae. In AD, dysbiosis is characterized by marked loss of microbial diversity and beneficial commensals, together with enrichment of pro-inflammatory taxa, including *Proteobacteria* and *Fusobacteriota*. Collectively, these microbial alterations are associated with reduced SCFA production, impaired intestinal barrier function, immune dysregulation, systemic inflammation and neurodegeneration, illustrating the progressive loss of microbial resilience proposed within the GBHC. Because reported microbial signatures vary across studies, they should be interpreted in the context of differences in sequencing methodologies, geographical origin, dietary habits, medication use and host genetics.

Current microbiome studies nevertheless present important limitations. Most rely exclusively on fecal samples, which provide only a partial representation of microbial communities throughout the GIT and poorly reflect the small intestine, despite its major roles in nutrient absorption, immune regulation and host–microbiota interactions. Emerging approaches enabling *in vivo* sampling of the small-intestinal microbiota may therefore identify alterations that occur before fecal dysbiosis becomes detectable (Tronel et al., 2025). In addition, most investigations have focused on bacteria, whereas fungal and viral communities remain comparatively unexplored. Although overall fungal diversity appears relatively preserved in AD, taxonomic shifts involving species such as *Candida tropicalis* and *Schizophyllum commune* suggest that the gut mycobiome may represent an additional component of AD-related gut ecosystem changes (Ling et al., 2021).

Alterations in microbial diversity, composition and ecological stability may reduce the capacity of the gut ecosystem to buffer subsequent perturbations. Within the GBHC framework, this ecological transition could contribute to chronic inflammation, barrier dysfunction and altered inter-organ communication, thereby increasing vulnerability to the putative biological tipping point described above.

## 4 Progressive disruption of homeostatic regulation within the GBHC

The mechanisms discussed below differ in their level of experimental and clinical support. Several biological processes incorporated into the GBHC, including barrier dysfunction, neuroimmune activation, altered metabolic signaling and impaired proteostasis, are supported by substantial experimental and human evidence. However, the proposition that these mechanisms become progressively coupled through systemic feedback loops and converge toward a critical tipping point remains a conceptual hypothesis (Figure 5). The GBHC therefore does not assume a unidirectional causal sequence from intestinal dysfunction to AD. Rather, it proposes that bidirectional interactions among peripheral and central systems may progressively alter the capacity to maintain homeostasis, with the relative contribution of each component varying according to disease stage, genetic background and individual biological context.

### 4.1 Barrier dysfunction

As adaptive capacity declines, dysfunction of these barriers may emerge or worsen. Persistent dysbiosis, chronic immune activation and metabolic stress can remodel the intestinal epithelium through changes in mucus composition, epithelial renewal and tight-junction architecture, leading to increased intestinal permeability (Manocha et al., 2019). Experimental AD models have demonstrated loss of junctional proteins, together with structural alterations of villi, crypts, Paneth cells and goblet cells (Lei et al., 2025; Palermo et al., 2025). These changes may facilitate the passage of microbial products, bEVs, nucleic acids and inflammatory mediators into the circulation.

The BBB undergoes similar progressive deterioration. Aging, vascular dysfunction and persistent inflammation impair endothelial integrity, reduce tight-junction expression and promote pericyte degeneration, thereby increasing BBB permeability (Sweeney et al., 2018; Banks et al., 2021; Knox et al., 2022; Nehra et al., 2022). Experimental and observational evidence suggests that dysfunction of one barrier may influence the other through reciprocal immune, metabolic and vascular interactions, although the direction and magnitude of these effects remain incompletely defined. Altered compartmentalization may therefore increase the opportunity for molecular and inflammatory signals originating in one tissue to influence distant compartments.

### 4.2 Chronic inflammation and neuroimmune dysregulation

Once barrier integrity is compromised, chronic low-grade inflammation may act as a convergent integrator of pathological processes within the GBHC. Unlike acute inflammation, which promotes tissue repair, persistent inflammatory activation can sustain cytokine production, oxidative stress and endothelial dysfunction, thereby further compromising tissue homeostasis.

The endotoxin hypothesis illustrates one possible component of this process. Experimental studies indicate that endotoxin exposure can induce systemic and neuroinflammatory responses, whereas human studies have reported associations between circulating endotoxin-related signals, inflammation and cognitive impairment. Increased intestinal permeability may facilitate exposure to circulating LPS and other microbial products. Elevated LPS has been reported in AD brain tissue and associated with amyloid burden and cognitive impairment, together with increased TNF-α, IL-6, IL-17A and IL-1β (Nehra et al., 2022; Böckels et al., 2026). These observations do not establish that gut-derived LPS is a primary driver of neurodegeneration in humans.

Microglial and astrocytic responses provide an additional interface between systemic perturbations and cerebral homeostasis. Their roles in synaptic maintenance, inflammatory regulation and responses to pathological proteins are well established. Peripheral inflammatory and metabolic signals can modify glial states through pattern-recognition receptors, including TLR2, TLR4 and CD14, and through cytokine-signaling pathways. Activated microglia can release inflammatory mediators, complement components such as C1q and C3, and reactive species that affect synaptic function, vascular integrity and neuronal homeostasis. Experimental studies further suggest that activated microglia can promote astrocytic states with neurotoxic or synapse-disrupting properties, sometimes referred to as A1-like phenotypes (Liddelow et al., 2017; Mary et al., 2024; Lei et al., 2025; Xu and Lu, 2025). Reactive astrocytes, characterized by increased GFAP expression, accumulate around amyloid plaques and neurofibrillary tangles and may reinforce local inflammatory responses (Saito and Saido, 2018). These interacting glial responses may contribute to Aβ aggregation, tau hyperphosphorylation, oxidative stress, synaptic dysfunction and neuronal loss, potentially aggravating the dysregulation of cerebral homeostasis (Vaziri et al., 2025).

Chronic inflammation may also be reinforced by functional remodeling of the gut ecosystem. Reduced availability of microbiota-derived SCFAs may alter epigenetic and immune-regulatory pathways, including those involved in regulatory T-cell differentiation and Th17 responses (Marizzoni et al., 2020; van Olst et al., 2021). As epithelial integrity becomes progressively compromised, increased exposure to PAMPs may sustain mucosal immune activation. Concurrent changes in microbial metabolism may reduce the production of immunoregulatory indole derivatives and redirect tryptophan metabolism towards the kynurenine pathway, generating metabolites associated with Aβ-related neurotoxicity (Yang et al., 2024). Alterations in bile acid-transforming microbial communities may affect the conversion of primary into secondary bile acids. In parallel, microbial production of trimethylamine from dietary precursors can contribute to circulating TMAO, a metabolite associated with vascular dysfunction and, in experimental AD models, with increased BACE1 expression (Nehra et al., 2022; Zhang et al., 2023).

Together, these changes may weaken colonization resistance and epithelial–microbial communication, thereby sustaining inflammatory activation across intestinal, peripheral and cerebral compartments. Their contribution to neurodegeneration is nevertheless likely to vary according to disease stage and individual biological context.

### 4.3 Breakdown of inter-organ communication

The disruption of biological barriers and persistence of inflammation may alter the communication networks that normally coordinate the GBHC. Neural, immune, metabolic and endocrine pathways may progressively shift from predominantly adaptive signaling toward maladaptive responses.

Alterations in the gut microbiota have been associated with changes in vagal and enteric neuronal function, potentially contributing to autonomic dysfunction, altered gastrointestinal motility and impaired neuroimmune regulation (Han et al., 2017, 2022; Liu et al., 2023). At the same time, persistent systemic inflammation activates circulating immune cells, whose cytokines influence distant organs, including the brain, where they may sustain microglial activation, affect amyloid clearance and reinforce neuroinflammatory responses (Kowalski and Mulak, 2019).

Metabolic and endocrine communication also becomes progressively dysregulated. Altered microbial metabolism reduces the production of metabolites generally associated with regulatory or homeostatic functions, including SCFAs, indole derivatives and secondary bile acids, while increasing metabolites associated in some studies with inflammatory or vascular dysfunction, such as TMAO and neurotoxic kynurenine metabolites (Kang et al., 2024). In parallel, chronic stress, insulin resistance and altered incretin signaling may impair metabolic homeostasis, vascular function and neuronal resilience (Nowell et al., 2023).

Within the GBHC, these changes may represent a transition from adaptive to maladaptive inter-organ signaling. Signals that normally support tissue integrity, immune tolerance and metabolic balance may instead reinforce barrier dysfunction, inflammation and proteostatic imbalance. The resulting reciprocal interactions could further reduce the capacity of interconnected compartments to restore homeostasis.

### 4.4 Impairment of physiological protein trafficking and systemic proteostasis

Under physiological conditions, brain-derived proteins can enter CSF and peripheral circulation through several interconnected clearance pathways, linking CNS proteostasis with peripheral elimination. Whether this connection constitutes a continuous brain-to-gut protein-clearance pathway remains to be established. As discussed in section “2 Building the Gut-Brain Homeostatic Continuum,” physiological protein trafficking involves the production, distribution and elimination of brain-derived proteins across interconnected compartments.

Increasing homeostatic stress may compromise this network. Reduced glymphatic drainage, BBB dysfunction and impaired endothelial transport can decrease the export of brain-associated protein species into the circulation (Peng et al., 2016). At the same time, systemic inflammation, vascular aging and metabolic dysfunction may affect peripheral protein clearance. Age- or disease-associated changes in transporter activity, including P-gp, may also influence Aβ efflux across the BBB (Chiu et al., 2015; Wei et al., 2016; van Olst et al., 2021). Whether comparable transporter-dependent mechanisms contribute to Aβ handling at the intestinal barrier remains unknown.

Although Aβ peptides have been detected in the intestinal lumen and feces (Wu et al., 2022; Pils et al., 2024, Seignobos et al., 2026), these studies did not establish their tissue of origin. Recent experimental observations are compatible with the possibility that CNS-associated protein signals may reach the GIT, but they do not yet establish a physiologically relevant brain-to-gut clearance pathway. Similarly, evidence that gut dysbiosis affects epithelial transport or microglial phagocytic activity does not by itself establish a direct impairment of peripheral brain-protein elimination.

If protein trafficking and elimination become inefficient, the balance between protein production, distribution, processing and clearance may be progressively disturbed. Changes in protein concentration, conformation, localization and molecular interactions could then favor synaptic dysfunction, aggregation and tissue injury. Soluble Aβ may contribute to antimicrobial defense by binding microbial cell-wall carbohydrates through a heparin-binding domain, thereby promoting microbial agglutination and entrapment, whereas tau stabilizes microtubules and supports axonal transport (Gosztyla et al., 2018; Kent et al., 2020; Prosswimmer et al., 2024). Under persistent inflammatory and proteostatic stress, however, these physiological functions may become associated with extracellular Aβ oligomerization, plaque formation and tau aggregation.

Gut dysbiosis may further accelerate this pathological transition. Functional bacterial amyloids produced by the intestinal microbiota have been shown to promote the aggregation of host amyloidogenic proteins through prion-like cross-seeding mechanisms while simultaneously sustaining innate immune activation, thereby amplifying neuroinflammation and potentially enhancing cerebral Aβ deposition (Kesika et al., 2021; Lei et al., 2025). Examples include curli (CsgA) produced by *Escherichia coli* and *Salmonella enterica*, TasA from *Bacillus subtilis* and FapC from *Pseudomonas aeruginosa* (Elkins et al., 2024). Some bacterial amyloids, including curli, may also act as PAMPs by engaging TLR2-containing receptor complexes with CD14 and activating NF-κB- and inflammasome-associated pathways (Rapsinski et al., 2015; Vaziri et al., 2025).

#### 4.4.1 Tau pathology follows a similar trajectory

Persistent neuroinflammation and chronic microglial activation promote tau hyperphosphorylation, aggregation and intercellular dissemination, notably through EVs carrying phosphorylated tau (Mary et al., 2024). Bacterial extracellular DNA and neurotoxic metabolites generated through the kynurenine pathway have also been implicated in tau pathology (Böckels et al., 2026). Conversely, restoration of a healthy gut microbiota attenuates both amyloid and tau pathology in transgenic AD models, supporting the existence of systemic mechanisms regulating brain proteostasis beyond the CNS (Kim et al., 2020). Although the reciprocal relationship between gut dysbiosis and tau pathology remains incompletely understood in humans, the association between increased fecal abundance of Lachnospiraceae and CSF phosphorylated tau positivity is consistent with this possibility (Verhaar et al., 2022; Bello-Corral et al., 2023).

Overall, these findings support the possibility that protein aggregation reflects disturbances in an integrated homeostatic network involving protein production, trafficking, processing and elimination. Within the GBHC hypothesis, Aβ and tau may act as biological amplifiers of an already vulnerable system, but their accumulation does not establish a single systemic clearance failure.

### 4.5 Extracellular vesicles: from homeostatic mediators to pathological propagators

As pathological stress increases, EV-mediated communication may change in vesicle abundance, cellular origin, cargo and recipient-cell responses. Both bEVs and hEVs may carry increased amounts of inflammatory mediators, regulatory RNAs or aggregation-prone proteins in specific disease contexts. Because EVs can interact with, and under certain conditions cross, biological barriers, they represent potential vehicles for molecular exchange between compartments. Whether this trafficking substantially contributes to AD progression in humans remains unresolved.

During dysbiosis, bEVs may transport PAMPs, bacterial amyloids, inflammatory mediators and regulatory RNAs that influence epithelial, endothelial, immune and glial cells. Conversely, brain-derived hEVs released by neurons and glial cells may carry Aβ, phosphorylated tau and inflammatory mediators into the circulation, consistent with the possibility of bidirectional pathological signaling (Yuyama and Igarashi, 2017; Brunello et al., 2020).

Extracellular vesicles may also participate in proteostasis failure. Encapsulation within membrane vesicles can protect Aβ oligomers, phosphorylated tau and other aggregation-prone proteins from extracellular degradation and facilitate their uptake by recipient cells. EV-associated pathological proteins may display enhanced seeding activity, promoting the propagation of protein misfolding between neighbouring and distant cells (Guo and Lee, 2014; Sardar Sinha et al., 2018; Brunello et al., 2020). EVs nevertheless remain physiological mediators of intercellular communication. Their potential pathological effects may therefore arise not from EV signalling itself, but from disease-associated changes in vesicle abundance, origin, cargo or recipient-cell responses. This dual role supports their investigation as both biomarkers of disease-related changes and potential targets for restoring homeostatic communication within the GBHC.

### 4.6 AD as a potential manifestation of progressive homeostatic dysregulation

The mechanisms described above suggest that dysfunction of biological barriers, immune regulation, inter-organ communication, protein trafficking and proteostasis may become increasingly interconnected during disease progression. Within the GBHC, these interactions are proposed as components of a potentially self-reinforcing network in which reduced adaptive capacity increases vulnerability to further perturbation.

This interpretation does not imply that all components fail simultaneously or follow a fixed temporal sequence. Available evidence instead indicates substantial bidirectional interactions and heterogeneity across individuals and disease stages. Gut dysbiosis, barrier dysfunction, chronic inflammation, altered EV signalling and proteostatic imbalance may therefore interact in different combinations and with different relative contributions. In this context, Aβ and phosphorylated tau may act as biological amplifiers of an already destabilized system.

The framework also raises the possibility that interventions directed at individual molecular pathways could be complemented by strategies targeting broader physiological determinants of disease progression. In addition to removing pathological proteins, future approaches might aim to restore the coordinated networks that support resilience across the GBHC.

Taken together, the mechanisms discussed in this section provide biological plausibility for the GBHC hypothesis but do not establish a single linear causal pathway from intestinal dysfunction to AD. The relative contribution of intestinal, vascular, immune, metabolic, vesicular and cerebral mechanisms is likely to vary across individuals and disease stages. The GBHC therefore offers a systems-level interpretation in which reciprocal interactions among these processes may progressively constrain physiological resilience and adaptive capacity.

The tipping-point concept should consequently be regarded as a theoretical construct rather than a demonstrated biological threshold in AD. Progressive accumulation of perturbations across interconnected systems could, in principle, exceed compensatory capacity and promote a transition from relatively compensated homeostasis toward self-reinforcing dysfunction. Whether such a threshold exists in human AD, whether the transition is gradual or abrupt, and whether it differs across individuals or disease subtypes remain open questions requiring longitudinal, mechanistic and experimental investigation.

## 5 Discussion and future perspectives

### 5.1 The Gut–Brain Homeostatic Continuum: a complementary framework

Over the past decades, remarkable advances have transformed Alzheimer’s disease (AD) research. The development of the AT(X)N framework, fluid biomarkers and disease-modifying therapies targeting amyloid pathology has profoundly transformed both diagnosis and clinical management (Jack et al., 2018). These advances have established that AD-related biological changes can be detected years, and in some cases potentially decades, before the onset of dementia. More recently, clinical trials of amyloid-targeting disease-modifying therapies have shown that modifying amyloid pathology can modestly slow clinical decline in selected patient populations (Long and Holtzman, 2019; Scheltens et al., 2021). At the same time, the marked heterogeneity of disease trajectories observed among individuals with comparable pathological burdens highlights the need for complementary perspectives extending beyond cerebral pathology alone.

The GBHC provides such a systems-level perspective by integrating the continuous interactions among the gut ecosystem, biological barriers, peripheral organs and the brain. Within this framework, AD is not considered the consequence of a single initiating event or a linear peripheral-to-central cascade. Rather, disease vulnerability and progression may emerge, at least in part, from the progressive dysregulation of coordinated physiological interactions across interconnected biological systems.

A central concept of the GBHC is therefore physiological resilience, defined here as the capacity of interconnected biological systems to maintain or restore functional homeostasis in the face of cumulative biological stress. Differences in metabolic, vascular, immune, intestinal and barrier function may influence this capacity and could contribute to the variability observed in clinical trajectories among individuals with similar amyloid or tau burdens.

This proposition remains to be tested. Although numerous studies have reported associations between alterations in the gut microbiome, immune and metabolic pathways, vascular function, barrier integrity and AD-related phenotypes, their temporal and causal relationships remain incompletely established in humans. In particular, peripheral dysfunction may represent an early determinant of vulnerability, a consequence of established cerebral pathology, or a dynamic component of a bidirectional process. The GBHC should therefore be regarded as an integrative, hypothesis-generating framework rather than as a new causal model of AD.

Future longitudinal and interventional studies will be required to determine whether changes in peripheral physiological function influence disease trajectories and whether such interventions can complement established disease-modifying therapies. Ideally, these studies should integrate central and peripheral measures over time to distinguish associations from causal relationships and early determinants of vulnerability from downstream consequences of disease.

### 5.2 Toward integrated therapeutic strategies

Disease-modifying therapies targeting amyloid, and potentially tau, represent major advances and are likely to remain important components of AD management (Cummings et al., 2023, 2024; Rabinovici et al., 2025). These approaches directly target established components of AD pathology and have provided important proof-of-concept that modifying disease biology can influence clinical progression.

The GBHC does not challenge this therapeutic paradigm. Instead, it raises the additional question of whether the physiological context in which disease-modifying therapies are administered may influence disease evolution and therapeutic response. If systemic resilience contributes to the capacity of the organism to tolerate or compensate for pathological stress, interventions aimed at preserving metabolic, vascular, immune, intestinal and barrier homeostasis could potentially complement treatments directed at specific molecular pathologies. This perspective is compatible with evidence supporting multidomain approaches to dementia prevention, in which several modifiable risk factors are addressed simultaneously (Kivipelto et al., 2020). Within the GBHC framework, complementary strategies could include lifestyle and nutritional interventions, optimization of metabolic and vascular health, management of factors associated with chronic inflammation and, where supported by evidence, interventions targeting gut physiology (Figure 7).

**FIGURE 7:**
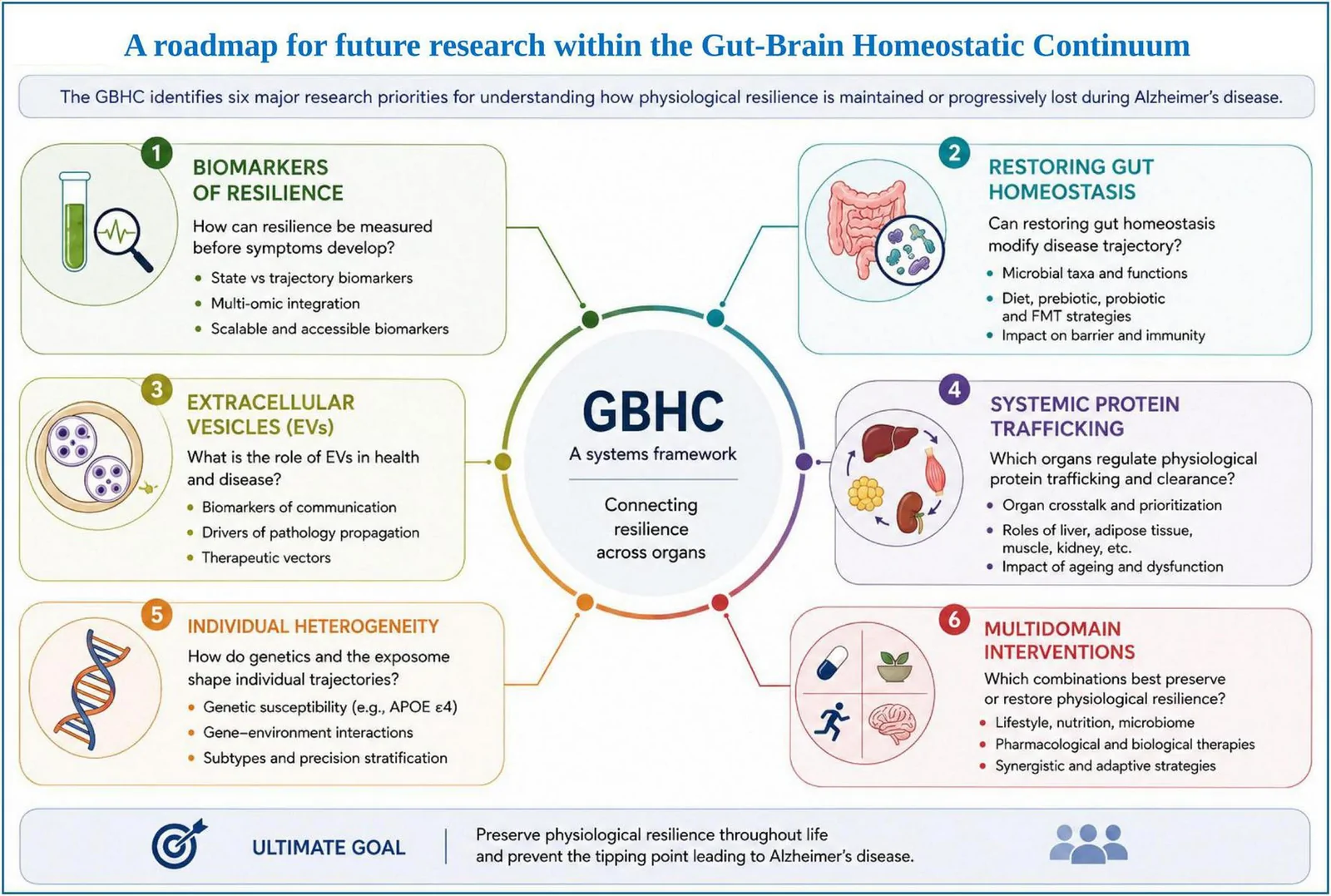
Future directions and research priorities for the GBHC. The GBHC proposes a shift from a disease-centered perspective to a resilience-centered framework in which AD emerges from the progressive failure of integrated gut-brain homeostasis. Six complementary research priorities are highlighted: (1) identifying biomarkers that quantify resilience rather than disease state; (2) restoring gut homeostasis by targeting the microbiota, intestinal barrier and host metabolism; (3) deciphering the role of EVs as mediators of inter-organ communication and physiological protein trafficking; (4) understanding the contribution of peripheral organs to physiological protein trafficking and clearance; (5) integrating genetic susceptibility, aging and environmental exposures to explain individual disease trajectories; and (6) developing multidomain interventions aimed at preserving physiological resilience throughout life. Collectively, these priorities provide a systems medicine roadmap for improving disease prediction, patient stratification and integrated therapeutic strategies designed to delay or prevent the biological tipping point leading to AD.

The GBHC does not imply that all biological pathways should be corrected simultaneously, nor that peripheral interventions should replace disease-modifying therapies. Rather, it suggests that preserving the interactions supporting physiological resilience may represent an additional objective alongside treatments targeting established AD pathology.

A major challenge will be to identify which components of systemic homeostasis are both biologically relevant and therapeutically modifiable. Future clinical trials should determine whether interventions targeting specific peripheral pathways affect validated AD biomarkers or clinical outcomes, and whether such effects are independent of, complementary to, or interactive with those of disease-modifying therapies.

### 5.3 From precision medicine toward systems medicine

Precision medicine has considerably improved the diagnosis and management of AD by integrating genetic background, fluid biomarkers and neuropathological signatures (Hampel et al., 2020; Bellenguez et al., 2022). These advances have made it possible to define increasingly precise biological subgroups and to identify individuals at different stages of disease. However, the substantial heterogeneity observed among individuals with similar central pathological profiles suggests that additional dimensions of biological variability may be relevant. The GBHC extends this perspective by proposing that clinically relevant heterogeneity may also reflect differences in the physiological state and adaptive capacity of interconnected peripheral and central systems. Individuals with similar amyloid or tau burdens may differ in microbial ecology, metabolic homeostasis, immune activation, vascular and barrier integrity, EV profiles or physiological protein trafficking. These dimensions may therefore provide complementary information for understanding inter-individual differences in disease progression and therapeutic response.

Established central biomarkers will remain essential for diagnosis, prognosis and clinical trial design. Future studies could, however, investigate multidimensional biomarker signatures combining central measures with indicators of peripheral physiology, including microbial composition and function, microbial metabolites, circulating inflammatory and metabolic markers, EV profiles, barrier integrity and processes related to physiological protein trafficking and clearance. Such signatures may help determine whether peripheral and central biomarkers provide independent, complementary or interacting information about disease trajectory and resilience.

Their clinical value remains to be established. Longitudinal studies will be particularly important to determine whether these markers reflect early determinants of vulnerability, consequences of established disease or dynamic responses to pathological stress. This temporal dimension will be necessary if resilience is to become a measurable biological concept rather than remain primarily theoretical.

EVs provide a particularly relevant example of a potential systems-level biomarker platform. Their molecular cargo reflects, at least in part, the physiological state of their cells of origin, and EVs can participate in communication between distant tissues (Kalluri and LeBleu, 2020; Welsh et al., 2024; Benameur et al., 2025; Da Costa et al., 2026). Their potential use as biomarkers and, in selected contexts, as therapeutic delivery vehicles warrants further investigation. However, the ability of EV-based approaches to capture integrated gut-brain physiology or predict individual disease trajectories remains to be demonstrated.

The systems medicine perspective does not require replacing established central biomarkers with peripheral measures. Rather, it proposes that combining complementary biological dimensions may provide a more comprehensive representation of disease heterogeneity. In this sense, the GBHC extends precision medicine toward systems medicine, in which central and peripheral information are considered together to investigate disease heterogeneity, identify potentially modifiable determinants of resilience and improve patient stratification.

### 5.4 Conclusion

The GBHC proposes a complementary systems-level framework in which AD is considered within the broader context of physiological resilience and its potential dysregulation across interconnected biological systems. By integrating the gut ecosystem, biological barriers, inter-organ communication, EVs and physiological protein trafficking, it provides a broader physiological context in which established mechanisms of AD can be investigated.

Importantly, the GBHC is not intended to replace current models of AD or to establish a new causal sequence linking peripheral dysfunction to neurodegeneration. Instead, it offers a hypothesis-generating framework for examining whether and how the physiological state of interconnected central and peripheral systems contributes to individual differences in vulnerability, resilience and disease progression.

A major priority for future research will be to establish the temporal relationships among peripheral and cerebral alterations. Longitudinal studies integrating central and peripheral biomarkers will be essential to determine whether changes in gut ecology, barrier integrity, immune and metabolic signaling, EV profiles or protein trafficking precede, accompany or follow AD-related cerebral pathology. Such studies should also account for the biological heterogeneity of AD and distinguish associations from causal relationships.

The translational value of the GBHC will ultimately depend on whether its conceptual propositions can be experimentally tested. This will require interdisciplinary approaches integrating neuroscience, gastroenterology, immunology, metabolism, microbiology and systems biology, together with longitudinal human studies and appropriately designed experimental models. Future research should also determine whether modifying selected components of systemic physiology can influence established AD biomarkers, clinical trajectories or responses to disease-modifying therapies.

The central question raised by the GBHC is therefore not whether peripheral systems cause Alzheimer’s disease, but whether the capacity of interconnected physiological systems to adapt to cumulative biological stress contributes to the resilience or vulnerability of the aging brain. Testing this proposition may provide new perspectives for understanding disease heterogeneity and identifying complementary strategies aimed at preserving physiological function throughout aging.
