# Supplementary material for: The Gut-Brain Homeostatic Continuum in Alzheimer’s disease: a systems framework for physiological resilience and disease progression
Source: Front Neurosci. 2026 Oct 19;20:1963040. doi: 10.3389/fnins.2026.1963040 (PMC13725227; doi:10.3389/fnins.2026.1963040)
Supplement: Supplementary file 1 [file Supplementary_file_1.docx]

| **(n)** | | | | |  |  |  |  |
| --- | --- | --- | --- | --- | --- | --- | --- | --- |
| **Control** | **SCD** | **MCI** | **aMCI** | **AD** | **Method** | **Key Taxonomic Findings** | **Geographic**  **Origin** | **Article** |
| 10 | — | — | — | 40 | qPCR | In amyloid+: ↓ *E. rectale*. ↑ *Escherichia/Shigella*. Strong link to inflammation | Italy | (Cattaneo et al., 2017) |
| 12 | — | — | — | 14 | 16S rRNA | AD: 5–10x higher bacterial load; ↑ *Actinomycetota* (*P. acnes*), ↑ *Bacillota*. **Analysis in brain post-mortem**. | UK | (Emery et al., 2017) |
| 25 | — | — | — | 25 | 16S rRNA | AD: ↓ *Bacillota*, ↓ *Bifidobacterium*, ↑ *Bacteroidota* | USA | (Vogt et al., 2017) |
| 4 | — | — | — | 4 | 16S rRNA | Isolation of cultivable butyrate producers. AD patients harbor viable SCFAs species | Japan | (Nguyen et al., 2018) |
| 43 | — | — | — | 43 | 16S rRNA | AD: ↓ *Bacteroidota*, ↑ *Actinomycetota*, ↑ *Ruminococcaceae* | China | (Zhuang et al., 2018) |
| 30 | — | 30 | — | 30 | 16S rRNA | MCI/AD: Similar alterations. ↑ *Escherichia*, *Lactobacillus*. ↓ *Bacteroides* | China | (Li et al., 2019) |
| 32 | — | — | 32 | 33 | 16S rRNA | aMCI: ↑ *Bacteroidota*. AD: ↓ Bacillota, ↑↑ *Enterobacteriaceae* | China | (Liu et al., 2019) |
| 60 | — | 24 | — | 24 | Shotgun | AD: ↑ *Klebsiella pneumoniae*. Dysregulation of anti-inflammatory P-glycoprotein pathway | USA | (Haran et al., 2019) |
| 94 | — | — | — | 34 | T-RFLP | Dementia: ↓ *Bacteroides* (Enterotype I), ↑ "Others" (Enterotype III) | Japan | (Saji et al., 2019) |
| 56 | — | — | — | — | 16S rRNA | APOE risk: APOE4 carriers show distinct microbial structure (↑ *Lachnospiraceae*) | UK | (Tran et al., 2019) |
| 38 | 53 | 8 | — | 6 | 16S rRNA | SCD: ↓ *Faecalibacterium* (progressive decline from Control to AD) | China | (Sheng et al., 2021) |
| 18 | — | 20 | — | 18 | 16S rRNA | MCI: ↓ *Lachnospira*. AD: ↓ *Bacteroides*, ↑ *Prevotella* | China | (Guo et al., 2021) |
| 34 | 26 | — | 18 | — | 16S rRNA | SCD: ↓ Alpha diversity. ↓ *Christensenellaceae*. aMCI: ↑ *Bacillota* | China | (M. Duan et al., 2021) |
| 71 | — | — | — | 100 | 16S rRNA | AD: ↓ *Faecalibacterium* (butyrate-producer). ↑ *Bifidobacterium* (lactate-producer) | China | (Ling et al., 2020) |
| 44 | — | — | — | 21 | 16S rRNA | Fecal markers (metabolites + bacteria) can discriminate AD from Controls with high accuracy | China | (Xi et al., 2021) |
| 47 | — | — | — | 30 | 16S rRNA | Compositional differences in Beta diversity despite no alpha diversity changes | China | (Hou et al., 2021) |
| — | 116 | 21 | — | 33 | 16S rRNA | In amyloid+: ↓ *Roseburia*, *Lachnospiraceae*. Association with p-tau status | Netherlands | (Verhaar et al., 2021) |
| 40 | — | — | — | 132 | 16S rRNA | ↑ *Fusobacteriota* and *Pseudomonadota* abundance with AD severity. ↓ *Bacillota* | China | (Chen et al., 2022) |
| 94 | — | 125 | — | 83 | 16S rRNA | MCI/AD: ↑ *Erysipelatoclostridiaceae* (increases with CDR). ↓ *Bacteroides* in AD | China | (Zhu et al., 2022) |
| 20 | — | 12 | — | 20 | 16S rRNA | AD: ↓ *Clostridiales* (incl. *Faecalibacterium*), ↑ *Escherichia*-*Shigella* | Thailand | (Wanapaisan et al., 2022) |
| 43 | — | — | — | 41 | 16S rRNA | AD: ↓ *Faecalibacterium*, ↓ *Bifidobacterium*, ↑ *Akkermansia* | Kazakhstan | (Cammann et al., 2023) |
| 115 | 49 | — | — | — | Shotgun | SCD amyloid+: ↑ *Dorea* *formicigenerans*, *Oscillibacter* *sp*. ↓ *Bacteroides faecis* | USA | (Ferreiro et al., 2023) |
| 13 | — | 38 | — | 34 | 16S rRNA | AD: ↑ *Clostridia*. ↓ *Blautia, Moryella*. Link to inflammatory mediators | Italy | (Marizzoni et al., 2023) |
| 208 | — | — | — | 24 | Shotgun | AD: ↓ *Bacillota*, ↓ *Clostridiaceae*, ↑ *Bacteroidota* | USA | (Kang et al., 2025) |

**Supplementary Table 1 Representative human clinical studies investigating gut microbiota composition across the AD continuum. Control** (Normal Control / Cognitively Unimpaired) subjects with healthy cognition (recent studies also verified amyloid-negative status via PET scan), **SCD** self-reported memory complaints but normal neuropsychological test scores, **MCI** measurable cognitive decline with preserved daily functioning, **aMCI** (amnestic MCI) MCI subtype with predominant episodic memory impairment, a stage most closely associated with AD, **AD** clinical dementia or biologically confirmed AD pathology. Microbial community profiling methods use **qPCR** with a "comparative Ct method" targeting candidate taxa, **16S rRNA sequencing** for bacterial taxa identification, **T-RFLP** for phylogenetic grouping analysis, and **shotgun** metagenomics for high-resolution species and functional profiling. Geographic origin is indicated, as microbiome composition can vary significantly based on regional dietary, environmental, and genetic factors.
